# Visible-Light Activation of Persulfate or H_2_O_2_ by Fe_2_O_3_/TiO_2_ Immobilized on Glass Support for Photocatalytic Removal of Amoxicillin: Mechanism, Transformation Products, and Toxicity Assessment

**DOI:** 10.3390/nano12234328

**Published:** 2022-12-05

**Authors:** Francis M. dela Rosa, Marin Popović, Josipa Papac Zjačić, Gabrijela Radić, Marijana Kraljić Roković, Marin Kovačić, María José Farré, Boštjan Genorio, Urška Lavrenčič Štangar, Hrvoje Kušić, Ana Lončarić Božić, Mira Petrović

**Affiliations:** 1 Faculty of Chemical Engineering and Technology, University of Zagreb, Marulićev trg 19, 10000 Zagreb, Croatia; 2 Catalan Institute for Water Research (ICRA), C/Emili Grahit, 101, 17003 Girona, Spain; 3 Faculty of Sciences, University of Girona, 17071 Girona, Spain; 4 Department of Safety and Protection, Karlovac University of Applied Sciences, Trg J.J. Strossmayera 9, 47000 Karlovac, Croatia; 5 Faculty of Chemistry and Chemical Technology, University of Ljubljana, Vecna pot 113, 1000 Ljubljana, Slovenia; 6 Catalan Institution for Research and Advanced Studies (ICREA), Passeig Lluis Companys 23, 08010 Barcelona, Spain

**Keywords:** Fe_2_O_3_/TiO_2_, amoxicillin, persulfate, H_2_O_2_, visible-light irradiation, transformation byproducts, toxicity

## Abstract

Fe_2_O_3_/TiO_2_ nanocomposites were fabricated via a facile impregnation/calcination technique employing different amounts iron (III) nitrate onto commercial TiO_2_ (P25 Aeroxide). The as-prepared Fe_2_O_3_/TiO_2_ nanocomposites were characterized by X-ray diffraction (XRD), Raman spectroscopy (RS), scanning electron microscopy/energy-dispersive spectroscopy (SEM/EDXS), X-ray photoelectron spectroscopy (XPS), Brunauer–Emmett–Teller analysis (BET), electron impedance spectroscopy (EIS), photoluminescence spectroscopy (PL), and diffuse reflectance spectroscopy (DRS). As a result, 5% (*w/w*) Fe_2_O_3_/TiO_2_ achieved the highest photocatalytic activity in the slurry system and was successfully immobilized on glass support. Photocatalytic activity under visible-light irradiation was assessed by treating pharmaceutical amoxicillin (AMX) in the presence and absence of additional oxidants: hydrogen peroxide (H_2_O_2_) and persulfate salts (PS). The influence of pH and PS concentration on AMX conversion rate was established by means of statistical planning and response surface modeling. Results revealed optimum conditions of [S_2_O_8_^2−^] = 1.873 mM and pH = 4.808; these were also utilized in presence of H_2_O_2_ instead of PS in long-term tests. The fastest AMX conversion possessing a zero-order rate constant of 1.51 × 10^−7^ M·min^−1^ was achieved with the photocatalysis + PS system. The AMX conversion pathway was established, and the evolution/conversion of formed intermediates was correlated with the changes in toxicity toward *Vibrio fischeri*. Reactive oxygen species (ROS) scavenging was also utilized to investigate the AMX conversion mechanism, revealing the major contribution of photogenerated *h*^+^ in all processes.

## 1. Introduction

Semiconductor-based photocatalysis has emerged as a promising technology for water purification. Among photocatalysts studied, titanium dioxide (TiO_2_) has been regarded as the “benchmark photocatalyst” due to its chemical and thermal stability, biological inertness, suitable mechanical properties, low cost, and nontoxicity [1,2,3]. However, TiO_2_ suffers from fast recombination of photogenerated charges (i.e., electron/hole pairs; *e^−^/h^+^*) and is active only under UV light due to its wide bandgap (3.0–3.2 eV), thus hindering its potential for visible-light-driven applications [1,4]. Consequently, these deficiencies can be improved by coupling TiO_2_ with a narrow-bandgap semiconductor with a visible-light response. As such, heterojunction formation between the two semiconductors promotes synergistic effects, leading to more efficient charge separation and improved photocatalytic activity under visible-light irradiation [2,4,5]. Hematite (α-Fe_2_O_3_) is a well-suited candidate for coupling with TiO_2_ due to its visible-light activity (bandgap energy (*E*_g_) = 1.9–2.2 eV) [6], natural abundance, low cost, and stability in a wide range of pH in aqueous solutions [7]. In recent years, the application of Fe_2_O_3_/TiO_2_-based composite photocatalysts has gained attention due to their efficiency for the removal of contaminants of emerging concern (CECs) from water [8]. Further photocatalytic activity enhancement can be accomplished using electron acceptors such as persulfate (S_2_O_8_^2−^, PS) and hydrogen peroxide (H_2_O_2_), which promote suppression of *e*^−^*/h^+^* recombination in Fe_2_O_3_/TiO_2_ photocatalysts, thus leading to the increased availability of photogenerated *h^+^* for the generation of additional reactive oxygen species (ROS) and for subsequent oxidation reactions [9,10].

However, several knowledge gaps need to be addressed regarding Fe_2_O_3_/TiO_2_ application in real environmental conditions, including the following:

(i) *Unequal distribution of solar energy around Earth’s surface.* Most applications of Fe_2_O_3_/TiO_2_ for CEC removal are reported under solar irradiation [8]. In fact, solar light is composed of 3% UV light, 44% visible light, and 53% infrared light [11,12]. The UV light portion within solar irradiation plays an important role for the activation of the overall photocatalytic composite (i.e., Fe_2_O_3_/TiO_2_), and its absence may result in different mechanisms [13]. It must also be noted that UV distribution at the Earth’s surface is unequal and influenced by several factors [14]. As such, focusing on the utilization of visible light is favorable for such environmental applications.

(ii) *Toxicity assessment*. Transformation and/or degradation byproducts of CECs after Fe_2_O_3_/TiO_2_ photocatalytic processes are scarcely reported. Clearly, the formation of more toxic byproducts has already been reported using different TiO_2_/semiconductor-based composites [15]. Accordingly, such potential treatment drawbacks must receive well-deserved attention since they can impose additional risk to the environment.

(iii) *Photocatalyst recovery*. Fe_2_O_3_/TiO_2_ immobilization in various supports is scarcely reported and is usually in powder form prior to the application. Immobilization of photocatalysts provides a potential decrease in operating costs for the water treatment process by avoiding in-treatment agglomeration and post-treatment separation issues [1,3,9,16,17].

Herein, the overall aim of the study was to provide insight into the visible-light activation of PS and H_2_O_2_ using immobilized Fe_2_O_3_/TiO_2_ for the photocatalytic removal of amoxicillin (AMX), a pharmaceutical that is included in the second EU “watch list” based on the proposed European Decision 2018/840/EU [18,19]. The specific goals of the present investigation were (i) to prepare Fe_2_O_3_/TiO_2_ via an impregnation/calcination technique, (ii) to characterize the as-prepared photocatalyst using instrumental techniques specific for the investigation of structural and elemental composition, as well as morphological, textural, optical, and electrochemical properties, (iii) to determine Fe_2_O_3_ content for optimal photocatalytic activity, (iv) to optimize the combined effect of pH and PS concentration for the removal of AMX using the immobilized Fe_2_O_3_/TiO_2_, and (v) to correlate AMX transformation byproducts under different photocatalytic processes (i.e., photocatalysis alone, photocatalysis + H_2_O_2_, and photocatalysis + PS) with each toxicity profile.

## 2. Materials and Methods

### 2.1. Materials

Amoxicillin (C_16_H_19_N_3_O_5_S, AMX, 96%, Acros Chemicals, Geel, Belgium) was used as a targeted contaminant of emerging concern. Aeroxide P25 (TiO_2_–P25, Evonik, Essen, Germany) and iron(III) nitrate nonahydrate (Fe(NO_3_)_3_·9H_2_O, ≥98.0%, Fluka, Buchs, Switzerland) were used as precursors for the preparation of nanocomposites. Ethanol (C_2_H_5_OH, EtOH, 96%, Gram-mol, Zagreb, Croatia), titanium isopropoxide (Ti{OCH(CH_3_)_2_}_4_, TTIP, 97%, Sigma-Aldrich, St. Louis, MO, USA), perchloric acid (HClO_4_, 70%, Kemika, Zagreb, Croatia), tetraethyl orthosilicate (Si(OC_2_H_5_)_4_, TEOS, 99%, Sigma-Aldrich, St. Louis, MO, USA), hydrochloric acid (HCl, 37%, Gram-mol, Zagreb, Croatia), and Levasil 200/30 (colloidal SiO_2_, Obermeier, Bad Berleburg, Germany) were used for the immobilization of as-prepared nanocomposites onto glass substrates. Formic acid (HCOOH, FA, HPLC grade, Sigma-Aldrich, St. Louis, MO, USA) and acetonitrile (CH_3_CN, HPLC grade, J.T. Baker, Philipsburg, NJ, USA) were used to prepare HPLC mobile phases. 2-Propanol ((CH_3_)_2_CHOH, p.a, Gram-mol, Zagreb, Croatia), Nafion (5 wt.%, Sigma-Aldrich, St. Louis, MO, USA), and sodium sulfate (Na_2_SO_4_, p.a., Kemika, Zagreb, Croatia) were used in photo electrochemical experiments. Sodium persulfate (Na_2_S_2_O_8_, PS, ≥99.0%, Sigma-Aldrich, St. Louis, MO, USA) and hydrogen peroxide (H_2_O_2_, 30%, T.T.T., Zagreb, Croatia) were used as oxidants. Sodium hydroxide (NaOH, p.a., Kemika, Zagreb, Croatia) and sulfuric acid (H_2_SO_4_, p.a., Kemika, Zagreb, Croatia) were used for pH adjustments. Methanol (CH_3_OH, MeOH, HPLC grade, J.T. Baker, Philipsburg, NJ, USA), tert-butanol ((CH_3_)_3_COH, *t*-BuOH, 99%, Lach-Ner, Neratovice, Czech Republic), FA (98%, Fluka, Buchs, Switzerland), and 1,4-benzoquinone (C_6_H_4_O_2_, BQ, 98%, Fluka, Buchs, Switzerland), were used for scavenging studies, i.e., for the determination of main species/mechanisms involved in degradation of AMX by the studied system. Throughout the study, all aqueous solutions were prepared with MilliQ-water, obtained using a Direct-Q3 UV Millipore (Merck, Burlington, MA, USA) ultrapure water system.

### 2.2. Preparation and Imobilization of Fe_2_O_3_/TiO_2_ Nanocomposites

The appropriate amount of TiO_2_–P25 (0.300 g) was dispersed in 80 mL of EtOH under sonication (Bandelin Sonorex RK 510 H, Berlin, Germany) for 5 min. Then, the appropriate amount of Fe(NO_3_)_3_·9H_2_O dissolved in 20 mL of EtOH was slowly added dropwise to the TiO_2_–P25 suspension whilst under sonication. After the sonication process was performed for 30 min, a brownish-white suspension was observed. The suspension was then continuously stirred for 6 h at room temperature, before drying at 60 °C for 12 h. The collected powder was calcined at 350 °C for 2 h in air using a muffle furnace (LP-08, Instrumentaria, Zagreb, Croatia) to obtain the final product. Different contents of Fe(NO_3_)_3_·9H_2_O were added to form final Fe_2_O_3_/TiO_2_ nanocomposites with a theoretical content (*w/w*) of 1%, 3%, 5%, 10%, and 20% (Fe_2_O_3_ to TiO_2_–P25). Pure α-Fe_2_O_3_ was obtained by performing the same procedure without the presence of TiO_2_–P25. Images of the prepared nanocomposites are shown in Figure S1.

The selected photocatalyst nanocomposites were immobilized using a low-temperature method [16]. The procedure involved the preparation of silica sol and titania sol. The silica sol was prepared via the hydrolysis of TEOS in water catalyzed by HCl, performed under vigorous stirring until a clear sol was obtained. Titania sol was prepared via the hydrolysis of TTIP in EtOH catalyzed by HClO_4_, conducted under reflux conditions at 100 °C for 48 h. Subsequently, the obtained silica sol, titania sol, EtOH, and Levasil 200/30 were mixed to form a binder sol in which 1.0 g of obtained photocatalyst was added. The mixture was homogenized in an ultrasonic bath for 10 min prior to the coating of round glass substrates (*r* = 37.5 mm) by spin coating at 1500 rpm for 30 s using a KW-4A spin coater (Chemat Technology, Los Angeles, CA, USA). The plates were thereafter heat-treated in an oven UN-55 (Memmert, Schawabach, Germany) at 200 °C for 2 h. The same procedure was repeated to prepare three catalyst layers, while the heating cycles (200 °C for 2 h) were applied between coatings of layers.

### 2.3. Characterization of Fe_2_O_3_/TiO_2_ Nanocomposites

X-ray diffractograms (XRD) of the prepared nanocomposites were recorded using an X-ray diffractometer MiniFlex 600 (Rigaku, Tokyo, Japan), using Cu Kα1 (λ = 1.54059 Å) radiation from 3° to 70° with a step width of 0.02° and scan speed of 2.00°/min.

Raman spectroscopy were measured using an Alpha300 (Oxford Instruments-Witec, Ulm, Germany) equipped with a microscope and attached atomic force microscope (AFM). The excitation source wavelength was set to 532 nm, while the integration time was set to 5 s with an average of 20 scans taken.

Scanning electron microscopy (SEM) images were obtained using an Ultra Plus SEM (Zeiss, Jena, Germany). Energy-dispersive spectroscopy spectra (EDS) were recorded with an X-max silicon drift detector (Oxford Instruments, Abingdon, UK).

X-ray photoelectron spectroscopy (XPS) measurements were performed using a PHI VersaProbe III (Version AD) (PHI, Chanhassen, MI, USA) equipped with a hemispherical analyzer and a monochromatic Al Kα X-ray source. Survey spectra were measured using a pass energy of 224 eV and step of 0.8 eV, while Fe *2p* core-level spectra were measured at a pass energy of 27 eV and step of 0.1 eV. The data were acquired using ESCApe 1.4 software. Fitting of Fe and Ti *2p* core-level spectra were performed using CasaXPS software.

Diffuse reflectance spectra (DRS) of the prepared nanocomposites were measured using a UV-2600i UV/Vis spectrophotometer (Shimadzu, Kyoto, Japan), equipped with an integrating sphere. The obtained reflectance versus wavelength spectra of pure components and nanocomposites were transformed into the Kubelka–Munk function (KM) versus photon energy (*hν*) in order to calculate bandgap (*E*_g_) values. The bandgap (*E*_g_) values of studied photocatalytic materials were calculated from the onsets of the absorption edge using the formula presented in Equation (1) [20].

(1) $$\lambda_{g}=\frac{1240}{E_{g}},$$

where *λ*_g_ is the bandgap wavelength.

Photoluminescence (PL) spectra were recorded at room temperature using a Varian Cary Eclipse fluorescence spectrophotometer (Agilent, Sta.Clara, CA, USA) with an excitation wavelength of 325 nm.

The Brunauer–Emmett–Teller (BET) single-point and multipoint surface area was determined from N_2_ adsorption/desorption isotherms using Gemini 2380 instrument (Micrometrics, Norcross, GA, USA). The nanocomposites were characterized in powdered form in all above-stated characterization techniques.

### 2.4. Photoelectrochemical (PEC) Measurements

Prepared nanocomposites were immobilized on 1 cm^2^ area of fluorine-doped tin oxide (FTO, Sigma-Aldrich, St. Louis, MO, USA) glass (2.2 mm thick; resistivity of 7 Ω/sq; overall dimension: 2 cm × 1 cm) using the method described by Elbakkay et al. [21]. Prior to coating, FTO glass slides were sonicated for 10 min sequentially in EtOH, acetone, and ultrapure water and then dried at room temperature. Thereafter, 2 mg of prepared nanocomposite was dispersed in 400 µL of 2-propanol and 10 µL of Nafion (Sigma-Aldrich, 5 wt.%) under sonication for 30 min. Finally, 30 µL of catalyst suspension was immediately drop-casted on 1 cm^2^ area of clean FTO glass and then dried in an oven at 80 °C for 30 min to form a working electrode.

Transient photocurrent responses and electrochemical impedance spectra (EIS) were obtained using a potentiostat/galvanostat PalmSens4 (PalmSensBV, Houten, The Netherlands) equipped with a standard three-electrode system and an LED light source (spectrum shown in Figure S2). Ag/AgCl electrode, Pt wire, as-prepared nanocomposite-coated FTO glass (1 cm^2^), and 0.1 M Na_2_SO_4_ solution were used as the reference electrode, counter electrode, working electrode, and electrolyte solution, respectively.

### 2.5. Photocatalytic Activity Evaluation

Photocatalytic treatment experiments with 0.05 mM AMX water solution were carried out in a water-jacketed (V = 0.09 L, T = 25.0 ± 0.2 °C) batch photoreactor illuminated by a simulated solar irradiation produced by Oriel Arc source (Newport; 450 W Xe lamp, Osram, Irvine, CA, USA), which was equipped with a collimator and airmass filter (AM 1.5 G), as well as an additional UV cutoff filter (λ > 400 nm) to provide only visible-light illumination [17]. In preliminary experiments a slurry system was used; 0.045 g of photocatalyst powder was dispersed with AMX solution (natural pH = 5.5) under constant stirring (300 rpm). The solution was continuously mixed for 30 min in the dark in order to achieve adsorption/desorption, denoted as (−30), and thereafter exposed to visible-light illumination. The onset of illumination is denoted as (0). During the experiments, 700 µL aliquots of samples were collected at designated time intervals (15, 30, 45, 60, 75, and 90 min), filtered through a 0.45 µM Chromafil XTRA RC (Macherey-Nagel, Duren, Germany) syringe filter, and immediately quenched with 100 µL of MeOH prior to HPLC analysis, as described in Section 2.6. The photocatalyst powder which possessed the highest photocatalytic activity was selected for immobilization onto glass plates as described in Section 2.2. The glass plates with immobilized photocatalytic material were placed at the bottom of the reactor in contact with AMX solution under constant mixing (90 rpm) by an orbital shaker DOS-20 (NeoLab, Heidelberg, Germany) and were subjected to a similar treatment procedure as described above for the slurry system, except for the illumination time intervals (15, 30, 45, 60, 75, 90, 120, and 150 min). A full factorial design (FFD) was utilized to study the effect of initial pH and PS concentration on AMX degradation (Table 1 and Table S1). The coded parameters *X*_1_ and *X*_2_ represent pH (ranging from 4 to 8) and concentration of PS (ranging from 500 µM to 3000 µM), respectively. The chosen minimum and maximum concentrations of PS corresponded to AMX:PS molar ratios of 1:10 to 1:60, respectively. The obtained optimal conditions for the degradation of AMX based on FFD experiments and response surface modeling were utilized as the basis for H_2_O_2_ conditions, which were later used and compared for the investigation of toxicity, transformation byproducts, and scavenging studies. Identification of reactive oxidizing species (ROS) was carried out using *t*-BuOH (5 mM), FA (5 mM), BQ (0.5 mM), and MeOH (5 mM) as scavengers for HO^•^, *h*^+^, O_2_^•−^, and both HO^•^ and sulfate radical (SO_4_^•−^), respectively. The experiments were conducted in triplicate, and average values are reported; the reproducibility of experiments was ≥95.5%.

### 2.6. Analytical Methods

pH measurements were performed using a Handylab pH/LF portable pH-meter (Schott Instruments GmbH, Mainz, Germany). AMX concentration was monitored using an HPLC, Series 10, (Shimadzu, Kyoto, Japan) equipped with a UV-DAD detector (SPD-M10A_VP_, Shimadzu) and a reversed-phase (RP) C18 column (250 mm × 4.6 mm, 5 μm, Macherey-Nagel Nucleosil, Duren, Germany). Isocratic elution was carried out with a mobile phase consisting of 90% aqueous 50 mM FA and 10% acetonitrile at an overall flow of 1 mL·min^−1^, whereas AMX was monitored at 272 nm. AMX transformation products (TPs) were analyzed using an ultrahigh-performance chromatograph (Thermo Scientific Vanquish system) in tandem with a high-resolution mass spectrometer (Orbitrap Exploris^TM^ 120, Thermo Scientific, Waltham, MA, USA), in positive and negative ionization mode. The samples were diluted fivefold with HPLC-grade water prior to the injection. Chromatographic separation of AMX and its transformation products was achieved on an RP C18 column (50 mm × 2.1 mm Hypersil GOLD^TM^, pore size 1.9 μm, Thermo Scientific, Vilnius, Lithuania). Gradient elution of water with 0.1% FA (A phase) and acetonitrile (B phase) was utilized, at a flow rate of 0.400 mL·min^−1^, under the following gradient program: 0–0.200 min, 2% B; 0.200–4.750 min, 98% B; 98% B maintained for 1.250 min (4.750–6.000 min); back to the initial mobile phase composition 3 min post run time (98% A/2% B). Ammonium acetate was used for negative mode instead of FA. The conditions for high-resolution mass spectrometry with an electrospray ionization source were the following: capillary, 3500 V; ion transfer tube temperature, 325 °C; vaporizer temperature, 350 °C; sheath gas pressure (Arb), 50; auxiliary gas pressure (Arb), 10; scan modes, full MS (resolution 60,000) and ddMS^2^ (resolution 15,000); scan range, *m*/*z* 100–1000. Raw MS data files of the control, blank matrix, and AMX samples were imported into Compound Discoverer^TM^ (v.3.3 SP1 Thermo Scientific, Waltham, MA, USA) software for transformation product identification. Fragment ion search (FISh) coverage function in Compound Discoverer^TM^ was utilized for structure elucidation and chemical transformations involved for each chromatographic peak. Expected compounds were measured within ±2 ppm of mass error; with maximum area ≥10^5^ and FISh coverage score ≥43.50. The aquatic toxicity of treated samples was evaluated using a commercial bioassay, based on inhibition of the luminescence emitted by Vibrio fischeri (VF) according to ISO 11348-3:2007 measured on a BiofixLumi-10 luminometer (Macherey-Nagel, Duren, Germany). Luminescence inhibition after 15 min exposure was taken as the endpoint. The results were expressed as effective concentrations causing a 50% reduction in bioluminescence (EC_50_) and converted into toxicity units (TU = 100/EC_50_).

### 2.7. Calculations

Response surface methodology (RSM) was utilized to determine the effectiveness of visible-light-driven photocatalytic treatment of AMX dependent on initial pH and PS concentration. The values of process parameters are represented by independent variables: *X*_1_ and *X*_2_ (Table 1). Experimental space was described using a 3^2^ full factorial design (FFD) for the vis-(5% Fe_2_O_3_/TiO_2_)/PS system, selected as the best according to preliminary results obtained in the slurry system (Table S1). The AMX conversion rate constants after a 150 min treatment period were chosen as process responses. The combined influence of studied parameters on process performance was described by a quadratic polynomial equation representing the RSM model, which was evaluated using a standard statistical test, i.e., analysis of variance (ANOVA), considering the following statistical parameters: Fisher *F*-test value (*F*), its probability value (*p*), regression coefficients (pure: *R*^2^; adjusted: *R*_adj_^2^), and *t*-test value. Moreover, graphical-based analysis was conducted on the so-called “residual diagnostic” (RD) using a normal probability test, Levene’s test, and a constant variance test. The calculations were performed using the Statistica 13.5 (Tibco, Palo Alto, CA, USA) and Design-Expert 10.0 (StatEase, Minneapolis, MN, USA) software packages.

## 3. Results and Discussion

### 3.1. Material Characterization

**Figure 1:**
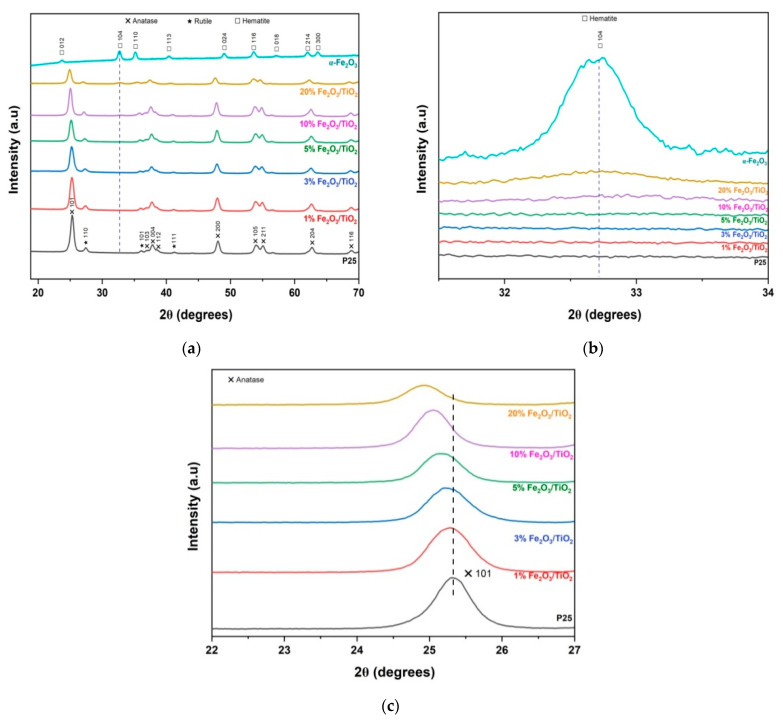
(**a**) XRD pattern of prepared Fe_2_O_3_/TiO_2_ nanocomposites; (**b**) partial magnification around (104) plane of hematite; (**c**) partial magnification around (101) plane of anatase.

The crystalline structures of the as-prepared photocatalytic materials were investigated using XRD. In Figure 1a, the peaks observed in the diffractograms at 2*θ* = 25.30°, 37.00°, 37.84°, 38.72°, 48.02°, 53.94°, 54.94°, 62.72°, and 68.92° were indexed into lattices (101), (103), (004), (112), (200), (105), (211), (204), and (116), respectively, which are in good agreement with anatase (ICDD PDF card 21-1272), while peaks at 2*θ* = 27.44°, 36.14°, and 41.22° were indexed into lattices (110), (101), and (111), respectively, which correspond to rutile (ICDD PDF card 21-1276) [22,23]. Meanwhile, preparation using only pure iron precursor yielded diffractogram peaks at 2*θ* = 23.72°, 32.74°, 35.20°, 40.40°, 49.06°, 53.02°, 57.10°, 62.08°, and 63.62°, which were indexed into lattices (012), (104), (110), (113), (024), (116), (018), (214), and (300), corresponding to pure hematite (ICDD PDF card 33-0664) [24,25]. Partial magnification around the (104) plane (Figure 1b) of hematite revealed that only 20% (*w/w*) Fe_2_O_3_/ TiO_2_ provided a noticeable additional peak, confirming the successful inclusion of α-Fe_2_O_3_, while no traces of hematite were detected in the remaining nanocomposites due to XRD detection limits [23]. In Figure 1c, partial magnification around 25.30° ((101), anatase plane), revealed a peak shift to a lower angle upon increasing the addition of Fe_2_O_3_, which is attributed to lattice distortion on the TiO_2_ surface [23].

**Figure 2:**
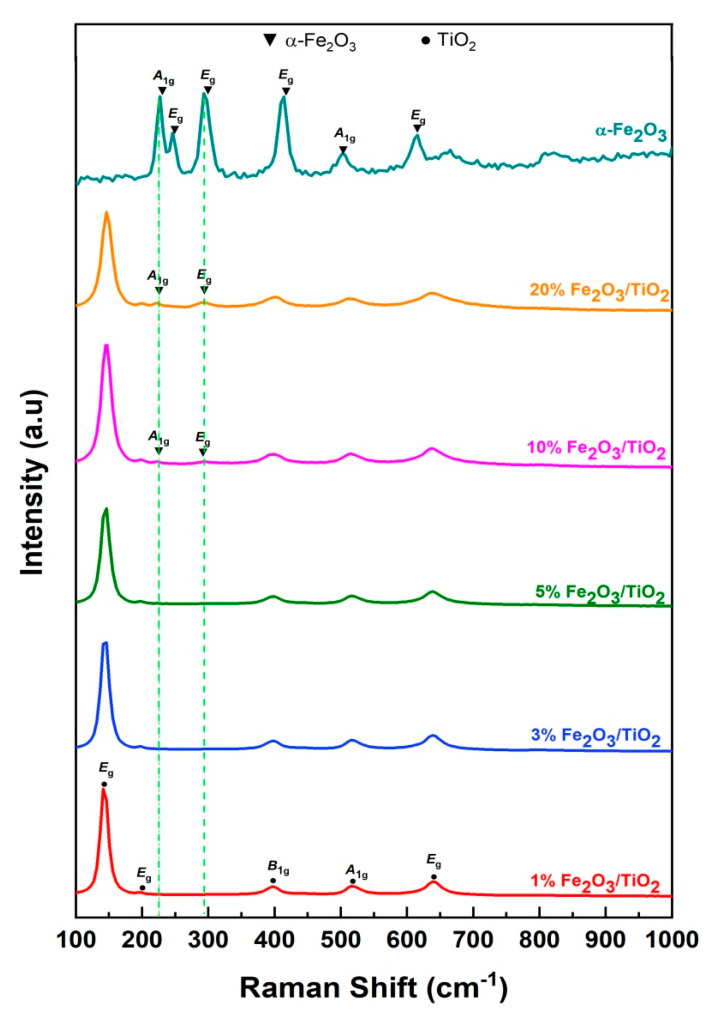
Raman spectra of Fe_2_O_3_/TiO_2_ nanocomposites and pure α−Fe_2_O_3_.

Raman spectra of the prepared nanocomposites and pure α-Fe_2_O_3_ are shown in Figure 2. All of the prepared nanocomposites showed distinct phonon modes of TiO_2_ such as *E*_g_ (143, 196, and 641 cm^−1^), *A*_1g_ (516 cm^−1^), and *B*_1g_ (396 cm^−1^) [26,27]. Meanwhile, α-Fe_2_O_3_ showed two *A*_1g_ phonon modes (227 and 496 cm^−1^) and four *E*_g_ phonon modes (245, 294, 410, and 613 cm^−1^) [24,28,29,30,31]. No vibrational modes of other iron-related species (i.e., maghemite or magnetite) were detected, which indicates the high purity of the obtained α-Fe_2_O_3_. It must be noted that only 10% and 20% (*w/w*) Fe_2_O_3_/TiO_2_ provided noticeable α-Fe_2_O_3_ vibrational modes (*A*_1g_ (227 cm^−1^), *E*_g_ (294 cm^−1^)), confirming the successful inclusion of α-Fe_2_O_3_ in the composite, which is also in agreement with the XRD results.

**Figure 3:**
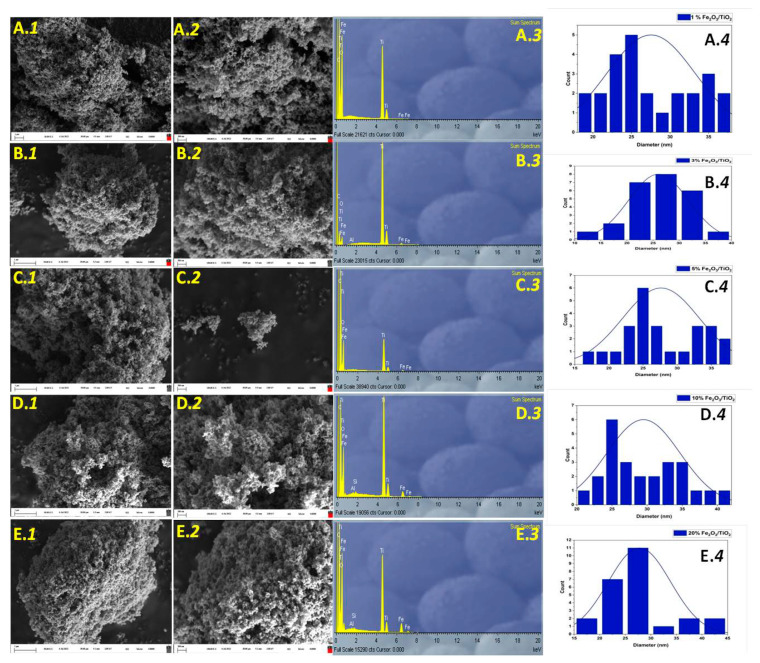
SEM images (50,000× (1); 100,000× (2); EDX spectrum (3); particle size distribution (4)) of (**A**) 1% Fe_2_O_3_/TiO_2_, (**B**) 3% Fe_2_O_3_/TiO_2_, (**C**) 5% Fe_2_O_3_/TiO_2_, (**D**) 10% Fe_2_O_3_/TiO_2_, and (**E**) 20% Fe_2_O_3_/TiO_2_ nanocomposites.

Scanning electron microscopy (SEM) images and EDX spectra of the prepared nanocomposite photocatalysts are shown in Figure 3. The formation of agglomerated TiO_2_–P25 (Aeroxide) particles is a consequence of the impregnation/calcination method. It must be noted that Fe_2_O_3_ content loading was low and did not cause any distortion of the overall appearance of the nanocomposite. As such, it can be derived that small Fe_2_O_3_ particles were formed around TiO_2_–P25 to promote a heterojunction between the semiconductors (i.e., TiO_2_ and Fe_2_O_3_), which may improve charge transfer mobility in the overall nanocomposite [23]. EDX spectra revealed the presence of small Fe amount among the prepared nanocomposites, which later proved the incorporation of Fe_2_O_3_. These results are in agreement with the obtained XRD and Raman results, as discussed above.

**Figure 4:**
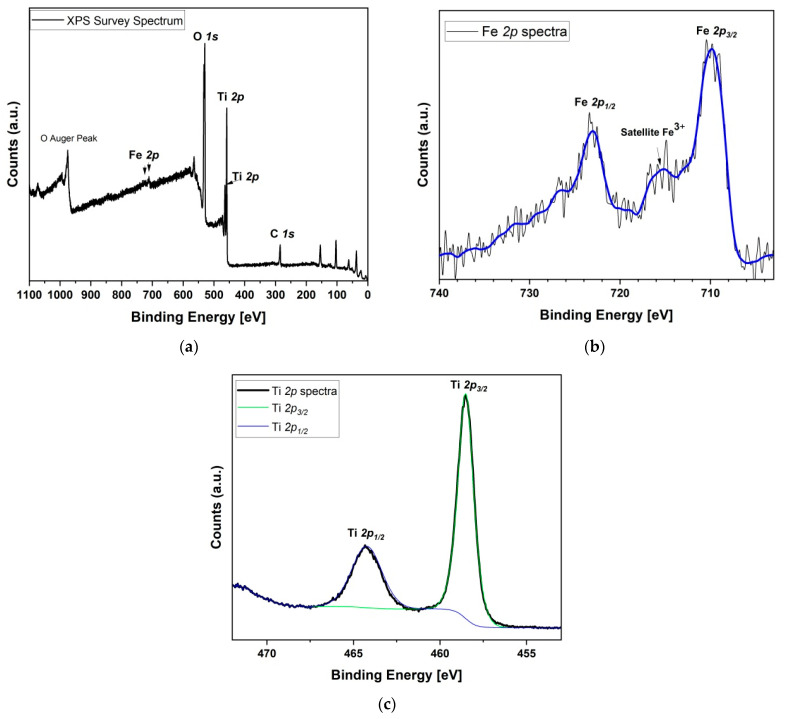
(**a**) XPS survey analysis of 5% Fe_2_O_3_/TiO_2_; (**b**) Fe 2p core-level spectrum; (**c**) Ti 2p core-level spectrum.

X-ray photoelectron spectroscopy (XPS) was further used to determine the surface chemical composition and oxidation states of 5% Fe_2_O_3_/TiO_2_ nanocomposites. The XPS full survey spectrum (Figure 4a) showed distinct signals of Fe 2p, Ti 2p, and O 1s, confirming the successful inclusion of α-Fe_2_O_3_ on the surface of TiO_2_ [32], while the C 1s peak was attributed to adventitious carbon contamination originating from air exposure of the samples [33]. In Figure 4b, the core-level XPS spectrum of Fe 2p showed two peaks at binding energy (BE) values of 723.50 and 709.85 eV, corresponding to Fe 2p_1/2_ and Fe 2p_3/2_, respectively, and a satellite signal at around 715 eV, which are all characteristic of Fe^3+^ in Fe_2_O_3_ [23,32,34]. Moreover, the difference in core energy level of Fe 2p, Δ(BE) = (2p_1/2_ − 2p_3/2_) = 13.65 eV also proved the presence to α-Fe_2_O_3_ [32,34]. In Figure 4c, the core-level XPS spectrum of Ti 2p showed Ti^4+^ characteristic peaks at BE values of 464.33 and 458.53 eV, corresponding to Ti 2p_1/2_ and Ti 2p_3/2_, respectively [23,32]. Similarly, Ti 2p, Δ(BE) = (2p_1/2_ − 2p_3/2_) = 5.8 eV, indicated the normal state of Ti^4+^ in TiO_2_–anatase, which is similar to the results reported in the literature [33,35,36].

**Figure 5:**
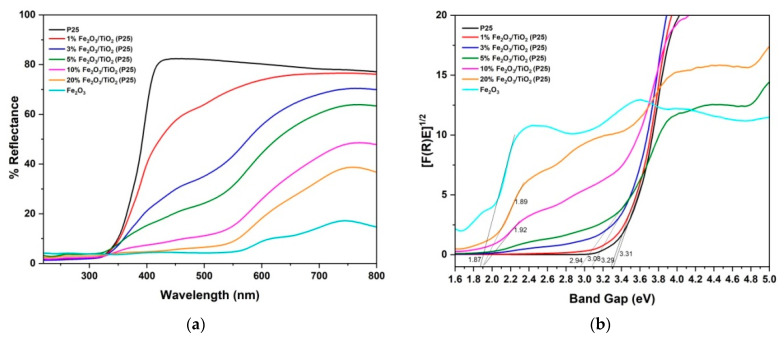
(**a**) UV/visible DRS spectrum of Fe_2_O_3_/ TiO_2_ nanocomposites; (**b**) Kubelka−Munk functions [F(R)E]^1/2^ versus photon energy (eV).

The UV diffuse reflectance spectra of pure components and prepared nanocomposites are shown in Figure 5a, whereas the Kubelka–Munk transformed spectra for the calculation of bandgap values are presented in Figure 5b. As shown in Table 2, calculated bandgap values of TiO_2_–P25 and α−Fe_2_O_3_ powders are in agreement with the values provided in the literature [37,38]. An increase in visible-light absorption (Figure 5a) and an overall decrease in bandgap values (Table 2) of the Fe_2_O_3_/TiO_2_ nanocomposites were observed upon increasing Fe_2_O_3_ content.

**Figure 6:**
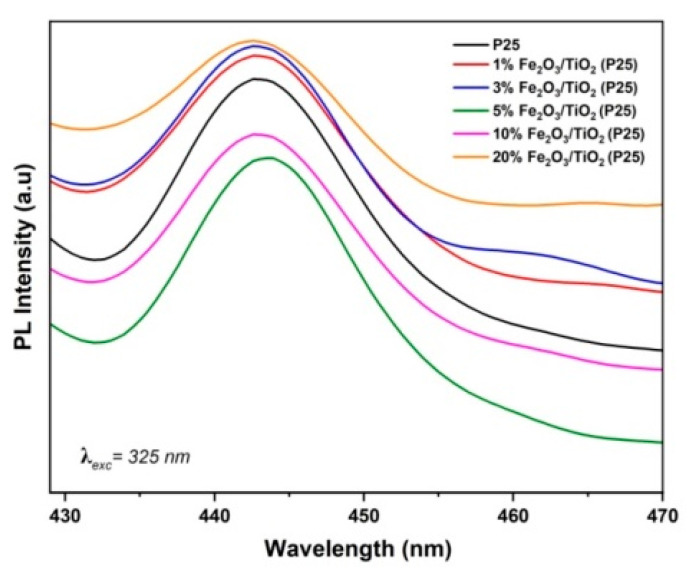
Photoluminescence (PL) spectra of pristine TiO_2_ and Fe_2_O_3_/ TiO_2_ nanocomposites.

Photoluminescence (PL) spectroscopy was used to study the separation of photogenerated *e*^−^/*h*^+^ pairs in the as-prepared nanocomposites. As can be seen in Figure 6, all Fe_2_O_3_/TiO_2_ nanocomposites showed a specific emission peak at around 444 nm, as similarly reported by Sayed et al. [39], albeit with different intensities. Materials containing 1% and 3% (*w/w*) Fe_2_O_3_ exhibited higher PL intensity compared to pristine TiO_2_. Such low Fe_2_O_3_ loading (i.e., 1 and 3% (*w/w*)) may suppress the defect concentration, thus promoting an increase in *e*^−^/*h*^+^ recombination rate [40,41]. Similarly, a further increase in Fe_2_O_3_ loading (i.e., 20% (*w/w*)) exhibited the highest PL intensity among all the prepared nanocomposites, higher than pristine TiO_2_. As such, an optimal level of 5% Fe_2_O_3_ loading exhibited the lowest PL intensity, suggesting a strongly suppressed *e*^−^/*h*^+^ recombination rate [42], which could be considered as having the highest photocatalytic activity among all the prepared nanocomposites.

**Figure 7:**
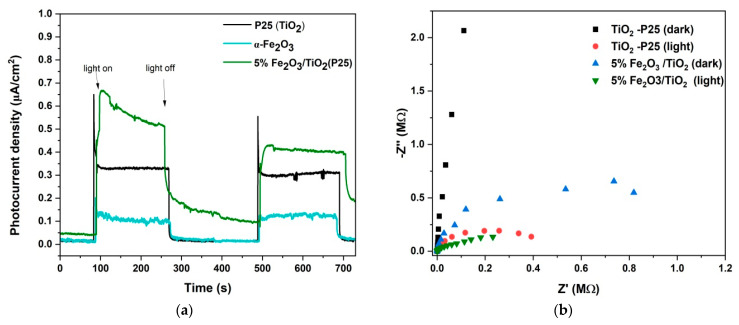
(**a**) Transient photocurrent responses of TiO_2_–P25, α−Fe_2_O_3_, and 5% Fe_2_O_3_/TiO_2_ nanocomposites; (**b**) EIS Nyquist plots of TiO_2_−P25 and 5% Fe_2_O_3_/TiO_2_ in dark and light conditions.

To further explore the photogenerated charge carrier separation efficiency of the prepared nanocomposite, photoelectrochemical studies (i.e., transient photocurrent responses and EIS) were conducted. The photocurrent density responses of a photocatalyst are directly related to its photocatalytic activity [43,44]. Transient photocurrent responses of TiO_2_, α−Fe_2_O_3_, and 5% Fe_2_O_3_/TiO_2_ are shown in Figure 7a. Specifically, 5% Fe_2_O_3_/TiO_2_ exhibited the highest response (0.55 μA·cm^2^) compared to individual parts of the composite (i.e., TiO_2_ and Fe_2_O_3_). The improved separation efficiency was attributed to successful heterojunction formation. It must be noted that the photocurrent density of 5% Fe_2_O_3_/TiO_2_ was reduced in the second cycle (light on/light off) to 0.45 μA·cm^2^, which may be attributed to the leaching of Fe_2_O_3_ [44]. Electron impedance spectroscopy (EIS) was used to study the interfacial charge transfer mechanism in the prepared samples [45]. As shown in Figure 7b, EIS Nyquist plots of pure TiO_2_ and 5% Fe_2_O_3_/TiO_2_ were measured under dark and light irradiation. In EIS, the radius of the semicircle corresponds to the overall charge transfer resistance [44,45,46]. Under visible-light irradiation, all samples showed less charge transfer resistance than in the dark, with 5% Fe_2_O_3_/TiO_2_ having a smaller radius than pure TiO_2_, indicating an efficient charge transfer mechanism between Fe_2_O_3_ and TiO_2_ due to successful heterojunction formation.

### 3.2. Photocatalytic Activity Tests

**Figure 8:**
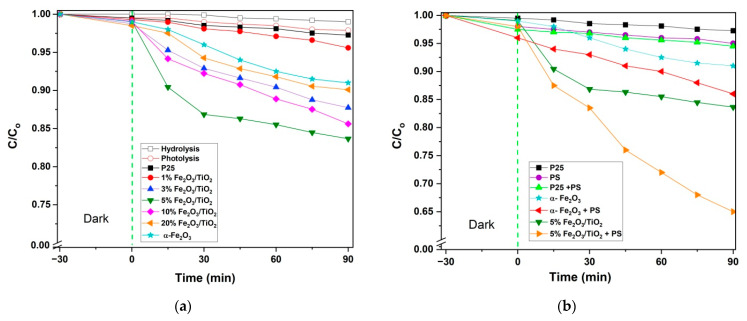
Photocatalytic removal of AMX using prepared photocatalysts under visible-light irradiation without oxidant (**a**,**b**) with [PS] = 0.3 mM. *Conditions:* [catalyst dosage] = 0.5 g/L; [AMX] = 0.05 mM; initial pH = natural pH (5.5); catalyst used in powdered form.

Preliminary experiments revealed a negligible effect of hydrolysis and photolysis on AMX concentration within the 90 min period (Figure 8a). Initial adsorption extents of AMX onto the prepared photocatalysts during the dark period (−30 to 0 min) were found to be infinitesimally small (<1.5%); thus, the observed removal extents of AMX during photocatalytic treatment were mainly approximated to the conversion extents. Such results were ascribed to the pKa values of AMX (pKa_1_ = 2.4, pKa_2_ = 7.4, and pKa_3_ = 9.6) [47] and the points of zero charge of TiO_2_–P25 (pH_PZC_ = 6.5–6.7) [48,49,50], α-Fe_2_O_3_ (pH_PZC_ = 6.2) [51], and Fe_2_O_3_/TiO_2_ (pH_PZC_ = 5.8–6.8) [39,52,53]. Hence, at pH 5.5, AMX is mostly present in its neutral for*m*/*z*witterionic form (pKa_1_ (2.4) < pH < pKa_2_ = 7.4 [47], and the net surface charge of all prepared photocatalysts is positive, thus leading to less interaction between two moieties. Single- and multipoint BET surface areas of the prepared photocatalysts are presented in Table 3. Incorporation of α-Fe_2_O_3_ with TiO_2_–P25 generally decreased the surface area of the prepared nanocomposites. However, such changes in surface area did not greatly affect much the adsorption behavior of the prepared photocatalysts since electrostatic interaction (i.e., pKa and pH_PZC_) played a major role in this scenario.

The highest photocatalytic activity was achieved by 5% Fe_2_O_3_/TiO_2_, exhibiting 16.3% AMX conversion within the 90 min period, which was significantly higher compared to any of the nanocomposites and pure components (i.e., TiO_2_–P25 and α-Fe_2_O_3_) (Figure 8a). Such an improvement in photocatalytic activity was ascribed to the suppression of recombination of photogenerated *e*^−^/*h*^+^ within the composite, as also proven and supported by PL spectroscopy (Figure 6) and photoelectrochemical experiments (Figure 7). Accordingly, 5% Fe_2_O_3_/TiO_2_ was selected as the photocatalyst to be immobilized onto glass support due to its superior photocatalytic activity to other prepared nanocomposites.

In Figure 8b, the presence of [PS] = 0.3 mM with 5% Fe_2_O_3_/TiO_2_ led to a significant increase in AMX conversion (35%). Such results are ascribed to additional SO_4_^•−^ (and potentially HO^•^) produced from PS, which serve as the electron acceptor and suppressor for *e*^−^/*h*^+^ recombination [9]. The determination of excess [PS] is shown in Figure S3. For further optimization, 5% Fe_2_O_3_/TiO_2_ was immobilized on the glass support (Figure S4), and RSM modeling was applied to avoid the obtention of misleading information from the conventional “one-parameter-at-time” approach [1]. As can be seen from Figure S5, i.e., the kinetic profiles of AMX conversions for the vis-(5% Fe_2_O_3_/TiO_2_)/PS system operated in conditions set by 3^2^ FFD (Table 1 and Table S1), the obtained results obeyed zero-order kinetics. Accordingly, AMX conversion rate constants (*k*obs) for the period of treatment under visible irradiation were calculated using Equation (2), representing the functional dependence of AMX conversion versus treatment time, implying a surface reaction mechanism for activation of PS [54,55,56]. Such calculated *k*_obs_ values were used as system responses in RSM.

(2) $$c_{0}-c=-k_{obs}\times t.\;$$

It must be noted that all photocatalytic experiments included a 30 min dark period to ensure adsorption/desorption equilibrium (Figure S5). For pH 4 and 6, the net surface charge of 5% Fe_2_O_3_/TiO_2_ was positive, while AMX mostly existed in neutral form; as a result, the absorbed amount of AMX was less than 1.5%, which is a consequence of less attraction between two moieties. For pH 8, it is expected that the absorbed amount of AMX would be less as well, since the net charges of 5% Fe_2_O_3_/TiO_2_ and AMX would both be negative, and repulsion of negative charges is expected to be dominant. However, AMX removal was observed to be 37–40% within the 30 min dark period, which can be associated with the base activation of persulfate [57,58]. In this case, the base-catalyzed hydrolysis of persulfate yields hydroperoxide anions and sulfate ions (Equation (3)). Thereafter, additional persulfate ion reacts with hydroperoxide anion to yield sulfate radicals and superoxide radicals (Equation (4)). Lastly, sulfate radicals can react with hydroxide ions to produce hydroxyl radicals (Equation (5)) [57,58]. Hence, it must be noted that the AMX removal associated with base-catalyzed persulfate was not included in RSM modeling since its process was characterized as a nonphotochemical reaction. As such, only the photocatalytic treatment (i.e., 0 to 150 min) was included, expressed as the AMX conversion rate constant, (kobs).

(3) $$S_{2}O_{8}^{\;2-}+2H_{2}O\;\overset{OH^{-}}{\to }\;2SO_{4}^{\;2-}+HOO^{-}+3H^{+}.\;$$

(4) $$S_{2}O_{8}^{\;2-}+HOO^{-}\;\to \;SO_{4}^{\;•-}+SO_{4}^{\;2-}+O_{2}^{\;•-}+H^{+}.$$

(5) $$SO_{4}^{\;•-}+OH^{-}\to OH•+SO_{4}^{\;2-}._{\;}$$

Accordingly, multiple regression analysis was applied on the FFD matrix and AMX (*k*obs) values calculated for the treatment period under visible-light irradiation (Table S1), yielding a polynomial equation for the RSM model, Equation (6).

*Y* = 1.41 − 0.2967 × *X*_1_ + 0.2467 × *X*_1_^2^ + 0.0433 × *X*_2_ − 0.1367 × *X*_2_^2^ + 0.0275 × *X*_1_ × *X*_2_. (6)

**Figure 9:**
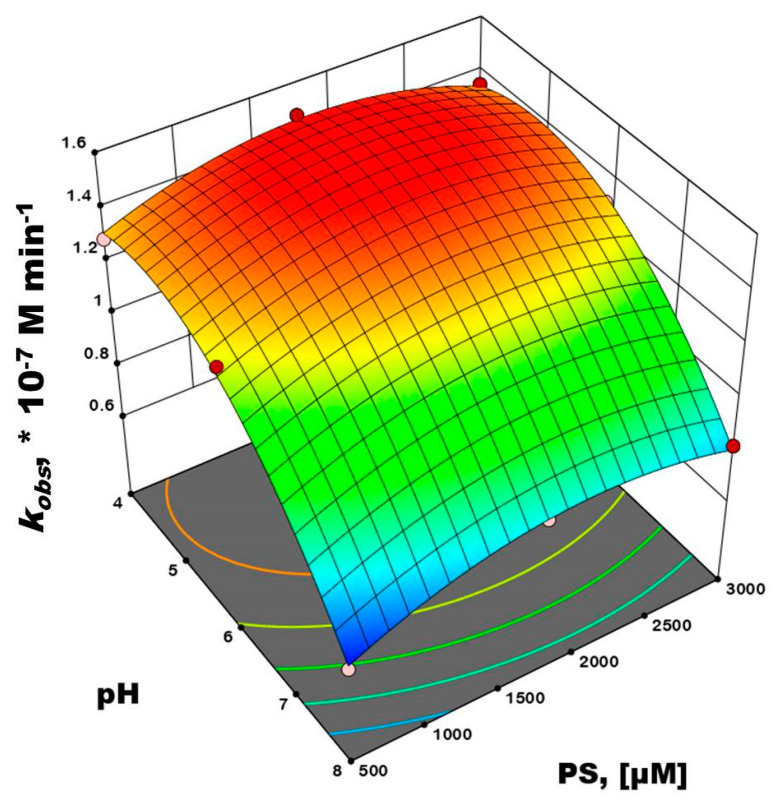
The 3D surface and contour plots presenting mutual interactions of initial pH and [PS] on photocatalytic AMX conversion by vis−(5% Fe_2_O_3_/TiO_2_)/PS (catalyst used in immobilized form).

The obtained model was characterized by ANOVA (Table S2) and RD tools (Figure S6), and it was found to be significant (*p* = 0.0010) and accurate (*R*^2^ = 0.9956 and *R*_adj_^2^ = 0.9883). On the other hand, RD revealed that (i) there were no violations in the assumptions that errors were normally distributed and independent of each other, (ii) the error variances were homogeneous, and (iii) the residuals were independent. ANOVA analysis also revealed that model terms corresponding to both process parameters (i.e., pH and [PS]) were significant, (*p* ≤ 0.05). (Table S2). Therefore, this model can be used as a tool to clearly discuss the influence of studied parameters on AMX conversion. The 3D surface and contour representations of the influence of initial pH and [PS] on AMX conversion rate (*k*obs), are shown in Figure 9.

As can be observed from Figure 9, an acidic pH (pH 4 to 6) was favorable for AMX conversion, which was associated with a high concentration of SO_4_^•−^ (*E*_o_ = 2.5–3.1 V vs. NHE), consisting of a higher oxidation potential than HO^•^ (*E*_o_ = 2.5–3.1 V vs. NHE) [59]. In addition, sulfate radicals are also dominant in acidic pH (pH 4 to 6) as described by Equations (7) and (8) [60,61]. An increase in pH toward basic range would lead to a decrease in the AMX conversion rate, which can be described by Equation (5) [62].

(7) $$S_{2}O_{8}^{\;2-}+H^{+}\to HS_{2}O_{8}^{-}.\;$$

(8) $$HS_{2}O_{8}^{-}\to SO_{4}^{\;•-}+HSO_{4}^{\;•}.$$

An increase in PS concentration was directly proportional to an enhancement of the AMX conversion rate up to the point where a further increase promoted a negative effect. Such a decrease in AMX conversion rate can be attributed to excess PS concentration, which promotes scavenging and terminates the formed radical species, as described in Equations (9)–(12) [63].

(9) $$S_{2}O_{8}^{\;2-}+SO_{4}^{\;•-}\to S_{2}O_{8}^{\;•-}+SO_{4}^{\;2-}.\;$$

(10) $$\;S_{2}O_{8}^{\;2-}+OH•\to HSO_{4}^{-}+SO_{4}^{\;•-}+0.5O_{2}^{\;}.$$

(11) $$\;SO_{4}^{\;•-}+SO_{4}^{\;•-}\to S_{2}O_{8}^{\;2-}._{\;}$$

(12) $$\;SO_{4}^{\;•-}+OH•\to HSO_{4}^{-}+0.5O_{2}^{\;}.$$

On the basis of the results presented in Figure 9, the optimum conditions for AMX conversion were pH 4.808 and a PS concentration of approximately 1873 μM, which were accurately calculated by maximizing the polynomial equation in Equation (6); thus, the predicted AMX conversion rate was 1.51 × 10^−7^ M·min^−1^. Accordingly, the obtained optimum conditions were further used as the basis for H_2_O_2_-assisted photoconversion experiments, which were later compared for the investigation of the AMX conversion mechanism, transformation byproducts, and toxicity studies.

**Figure 10:**
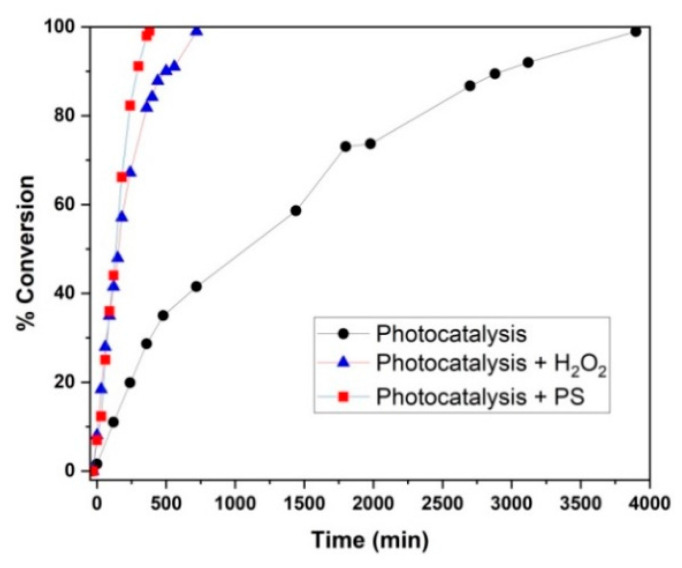
The %AMX conversion with photocatalysis, photocatalysis + H_2_O_2_, and photocatalysis + PS. Experimental Conditions: pH = 4.808; [PS] = [H_2_O_2_] = 1.873 mM; catalyst = immobilized 5% (*w/w*) Fe_2_O_3_/TiO_2_; [AMX] = 0.05 mM.

As shown in Figure 10, three photocatalytic processes (i.e., photocatalysis, photocatalysis + H_2_O_2_, and photocatalysis + PS) were compared on the basis of their AMX conversion profiles upon reaching <99%. Photocatalysis + PS was shown to be the fastest, reaching the full %AMX conversion within 380 min. Photocatalysis + H_2_O_2_ also showed improved full AMX conversion (within 720 min) compared to photocatalysis alone (3900 min). Photocatalysis only relies on photogenerated *h*^+^, O_2_^•−^, and HO^•^ as ROS for AMX conversion (Equations (13)–(16)). Accordingly, 5% Fe_2_O_3_/TiO_2_ can be excited using visible light to yield photogenerated *e*^−^/*h*^+^ (Equation (13)). Thereafter, photogenerated *e*^−^ reacts with O_2_ (dissolved in water) to form O_2_^•−^ (Equation (14)) [13,64,65]. Photogenerated *h*^+^ accumulated in the valence band (VB) of Fe_2_O_3_ may react with OH^−^ to form HO^•^ (Equation 15) [64], and photogenerated *h*^+^ may directly react with AMX (adsorbed at the catalyst surface), thereby producing transformation byproducts (Equation (16)).

(13) $$5\%\;Fe_{2}O_{3}^{\;}/TiO_{2}^{\;}+hv\;\left(visible\;light\right)\to e_{\;CB}^{-}+h_{\;VB}^{+}.$$

(14) $$e_{\;CB}^{-}+O_{2}^{\;}\to \;O_{2}^{\;•-}._{\;}$$

(15) $$h_{\;VB}^{+}+OH^{-}\to OH•._{\;}$$

(16) $$h_{\;VB}^{+}+AMX\to AMX\;\left(products\right)._{\;}$$

The improved AMX conversion via photocatalytic processes with oxidants can be ascribed the reactions of photogenerated *e*^−^ with H_2_O_2_ and PS to form HO^•^ and SO_4_^•−^, respectively (Equations (17) and (18)) [66].

(17) $$e_{\;CB}^{-}+H_{2}O_{2}^{\;}\to OH^{-}+OH•.$$

(18) $$e_{\;CB}^{-}+S_{2}O_{8}^{\;2-}\to SO_{4}^{\;2-}+SO_{4}^{\;•-}._{\;}$$

### 3.3. Mechanism

**Figure 11:**
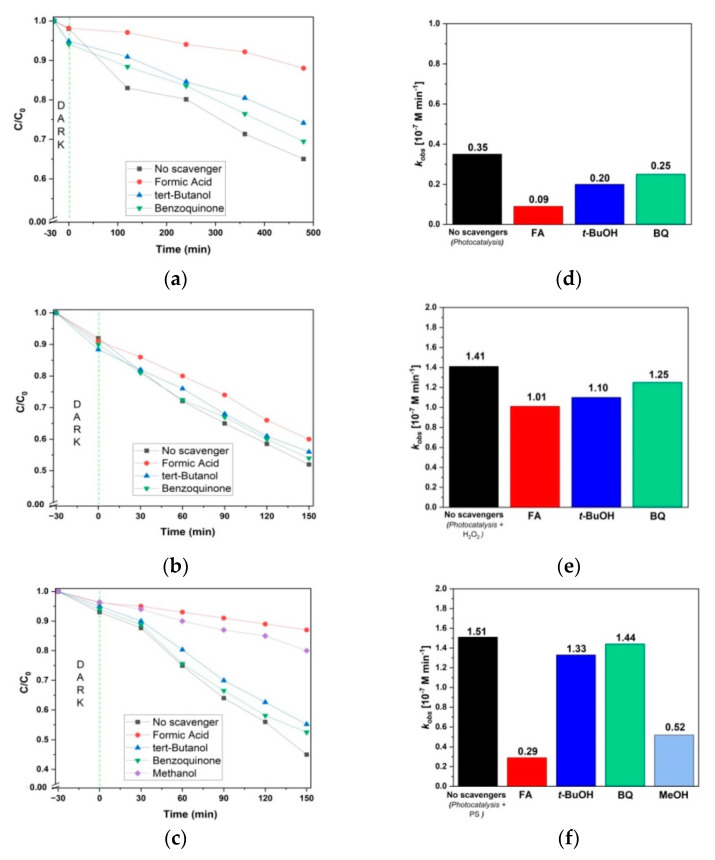
AMX conversion under photocatalysis (**a**), photocatalysis + H_2_O_2_ (**b**), and photocatalysis + PS (**c**) in presence of scavengers; (**d**–**f**) corresponding zero-order rate constants for each process. Experimental conditions: [AMX] = 0.05 mM; initial pH = 4.808; [PS] = [H_2_O_2_] = 1.873 mM; [FA] = [MeOH] = [*t*-BuOH] = 5 mM; [BQ] = 0.5 mM; catalyst used in immobilized form.

The AMX conversion mechanisms via photocatalysis, photocatalysis + H_2_O_2_, and photocatalysis + PS systems were studied in the presence of ROS scavengers (Figure 11). FA was used for scavenging photogenerated *h*^+^, while BQ was used to scavenge O_2_^•−^ (*k* = (0.9–1.0) × 10^9^ M^−1^·s^−1^) [67,68]. MeOH and *t*-BuOH were used to differentiate the contributions of SO_4_^•−^ and HO^•^. In such a case, MeOH reacts with both SO_4_^•−^ and HO^•^ (*k* = 1.1 × 10^7^ M^−1^·s^−1^ and *k* = 9.7 × 10^8^ M^−1^·s^−1^, respectively) [69,70]. Conversely, *t*-BuOH reacts three-orders-of-magnitude higher with HO^•^ (*k* = 9.7 × 10^8^ M^−1^·s^−1^, than with SO_4_^•−^ *k* = (4.0 − 9.1) × 10^5^ M^−1^·s^−1^ [66]), thus making *t*–BuOH as an efficient scavenger for HO^•^.

The AMX conversion and kinetic profiles achieved by photocatalysis in the presence of ROS scavengers are shown in Figure 11a,d, respectively. The highest inhibition of AMX conversion occurred in the presence of FA, resulting in only 12% AMX degradation (comparing to 35% obtained in the absence of any scavenger). This indicated that photogenerated *h*^+^ plays the main role in AMX photocatalytic conversion. Similarly, Zhu et al. reported that the Fe_2_O_3_–TiO_2_/fly ash cenosphere composite’s main active species for degradation of methylene blue were also photogenerated *h*^+^ [71]. Furthermore, it was observed that AMX conversion was reduced to 31% and 26% in the presence of BQ and *t*-BuOH, respectively. Such results indicated that HO^•^ plays a more significant role than O_2_^•−^. Hence, the order of ROS in decreasing contribution under the photocatalysis process is as follows: *h*^+^ > HO^•^ > O_2_^•−^.

The AMX conversion and kinetic profiles achieved by photocatalysis + H_2_O_2_ in the presence of ROS scavengers are shown in Figure 11b,e, respectively. The highest inhibition of AMX conversion occurred in presence of FA, resulting in an 8% reduction compared to the case without scavengers (40% and 48% AMX degradation, respectively). This indicates that photogenerated *h*^+^ plays a major role in AMX conversion. Similarly, Monteagudo et al. reported the dominant role of *h*^+^ in the solar–TiO_2_/H_2_O_2_ system for degradation of aniline [66]. AMX conversion in presence of *t*–BuOH was reduced to 44%. It is important to note that, even though *h*^+^ plays the major role, the HO^•^ contribution is nearly the same, as shown by the comparison of their rate constants (Figure 11e). Lastly, the presence of BQ reduced AMX conversion only to 46%, showing that superoxide radical plays a minor role in the overall process. Hence, the order of ROS in decreasing contribution in the photocatalysis + H_2_O_2_ process is as follows: *h*^+^ ≥ HO^•^ > O_2_^•−^.

The AMX conversion and kinetic profiles achieved with photocatalysis + PS in the presence of ROS scavengers are shown in Figure 11c,f, respectively. FA promotes the greatest inhibition among all scavengers used, yielding an AMX conversion of only 13% (compared to 55% in the case with no scavenger), implying that photogenerated *h*^+^ plays a major role in AMX conversion. Similar results were obtained upon performing persulfate activation-related processes such as solar/TiO_2_/S_2_O_8_^2−^ [63], solar/TiO_2_–Fe_2_O_3_/PS [9], and vis–TiO_2_/FeOCl/PS [72], which all reported that photogenerated *h*^+^ was the main oxidative species. On the other hand, AMX conversion was reduced to 20% and 45%, in the presence of MeOH and t–BuOH, respectively. Accordingly, SO_4_^•−^ plays a more significant role than HO^•^, as expected due to the acidic conditions applied. The presence of BQ resulted in rather low inhibition, up to 47.5% of AMX degraded, suggesting that O_2_^•−^ only contributes a minor role. Therefore, the overall order of ROS in decreasing contribution by photocatalysis + PS is as follows: h^+^ > SO_4_^•−^ > HO^•^ > O_2_^•−^.

**Figure 12:**
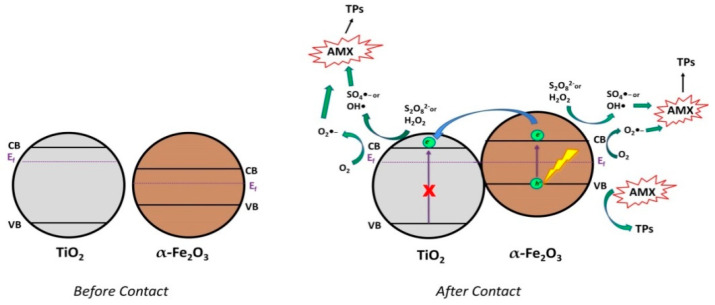
Proposed charge transfer mechanism between Fe_2_O_3_ and TiO_2_ heterojunction before and after contact, under visible−light irradiation.

The combined mechanism of the three photocatalytic systems is shown in Figure 12. The combination of TiO_2_ and Fe_2_O_3_ leads to the formation of a *Type 1 heterojunction* [5], where the valence band (VB) and conduction band (CB) of Fe_2_O_3_ are in between the VB and CB of TiO_2_, (Figure 12, *before contact*). However, such a heterojunction formation is unfavorable for the effective separation of photogenerated charges (*e*^−^/*h*^+^) due to the migration/accumulation to Fe_2_O_3_. Xia et al. [64], Liu et al. [65], and Mei et al. [44] proposed that, in order to achieve greater charge separation between Fe_2_O_3_ and TiO_2_, the fermi level of each semiconductor must be equalized. Thereafter, photogenerated electrons can flow from the CB of Fe_2_O_3_ to the CB of TiO_2_ under visible–light irradiation (Figure 12, *After Contact*). Additionally, photogenerated *e*^−^ can react with O_2_, H_2_O_2_, and S_2_O_8_^2−^, yielding O_2_^•−^, HO^•^, and SO_4_^•−^, respectively, while photogenerated holes react directly with AMX and HO^−^, forming HO^•^.

### 3.4. AMX Transformation Byproducts and Toxicity Evaluation

**Figure 13:**
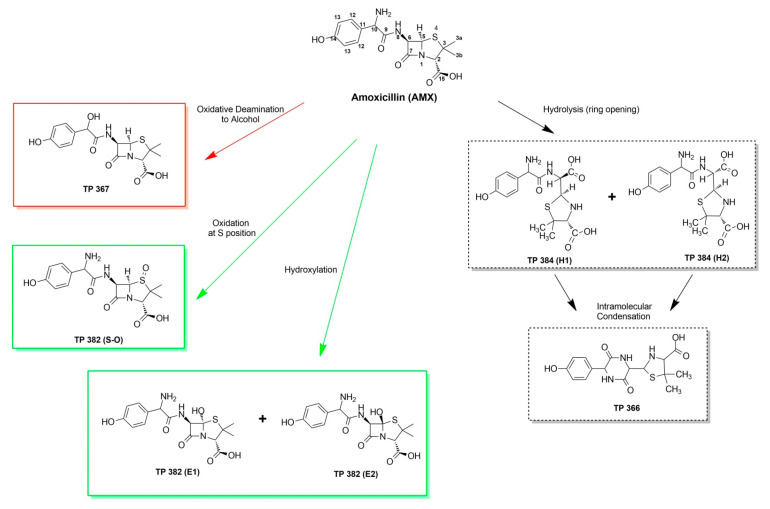
Proposed transformation byproducts of AMX under different photocatalytic treatment processes. Black boxes indicate the transformation byproducts obtained in all processes; red boxes indicate the transformation products obtained under photocatalysis and photocatalysis + H_2_O_2_; green boxes indicate the transformation by-products obtained under photocatalysis + H_2_O_2_ and photocatalysis + persulfate.

The transformation products (TPs) of AMX in photocatalysis, photocatalysis + H_2_O_2_, and photocatalysis + PS systems were investigated and identified using LC–HRMS-orbitrap in positive and negative modes. The TPs detected and their corresponding mass spectra are presented in Table S3 and Figures S7–S14, respectively. The annotated Δmass (error) between the experimental mass-to-charge ratio (*m*/*z*) and theoretical values (*m*/*z*) values of all proposed chemical formula was less than ± 2 ppm with an FISh coverage score ≥43.50, which allows accuracy in the assignment of elemental composition and fragment ion elucidation, respectively. It must be noted that only results from positive modes were elucidated, since all results from negative modes showed FISh coverage ≤40%. As shown in Figure 13, three TPs (TP 384 (H1), TP 384 (H2), and TP 366) were detected in all processes studied. TP 384 (H1) and TP 384 (H2) correspond to penicilloic acid (C_16_H_21_N_3_O_6_S) (i.e., the hydrolysis byproduct of AMX), which is formed via the reaction of H_2_O molecule with the strained four-membered *β*-lactam ring of AMX [73,74]. TP 366 corresponds to amoxicillin 2′,5′-diketopiperazine (C_16_H_19_N_3_O_5_S), which is formed via the loss of H_2_O and then further condensation of TP 384 (H1) or TP 384 (H2) [75].

TP 367 was detected in both photocatalysis and photocatalysis + H_2_O_2_ treatments, which can be attributed to two-step successive transformation (i.e., (1) oxidative deamination, and (2) reduction to alcohol) of AMX (Figure S15). Oxidative deamination byproducts formation of *β*-lactam derivatives is ascribed to the abstraction of α-hydrogen atoms, leading to the formation of a carbonyl derivative [76]. In such a case, the >CH-NH_2_ moiety of AMX can be transformed into an imine moiety >CH=NH; then, further cleavage of the carbon–nitrogen double bond occurs, yielding a C=O moiety, TP (*m*/*z*) = 365. However, it must be noted that the intermediate TP (*m*/*z*) = 365 was not detected in any of the photocatalytic processes studied since its carbonyl moiety is further reduced to alcohol, forming the detected derivative, TP 367. The involved reduction reaction may be attributed to photocatalytic hydrogenation of TP 365 with the assistance of AMX as a “self” hydrogen donor (H^+^) and sacrificial agent. Similarly, Wei et al. reported simultaneous hydrogen production and degradation of AMX using Bi spheres-g-C_3_N_4_ [77] and MoS_2_@Zn_x_Cd_1−x_S [78], supporting the assumption that persistent organic pollutants can be used as sacrificial electron donors. Conventionally, low C-atom alcohols (i.e., methanol, ethanol, isopropanol, triethanolamine, etc.) and low C-atom carboxylic acids (i.e., lactic acid) are used as sacrificial electron donors for photocatalytic hydrogenation and H_2_ production [5,79]. In this case, it can be assumed that AMX and its byproducts (i.e., low C-atom species) mimic the role of lower C-atom alcohols in photocatalytic hydrogenation/hydrogen-forming reactions.

**Figure 14:**
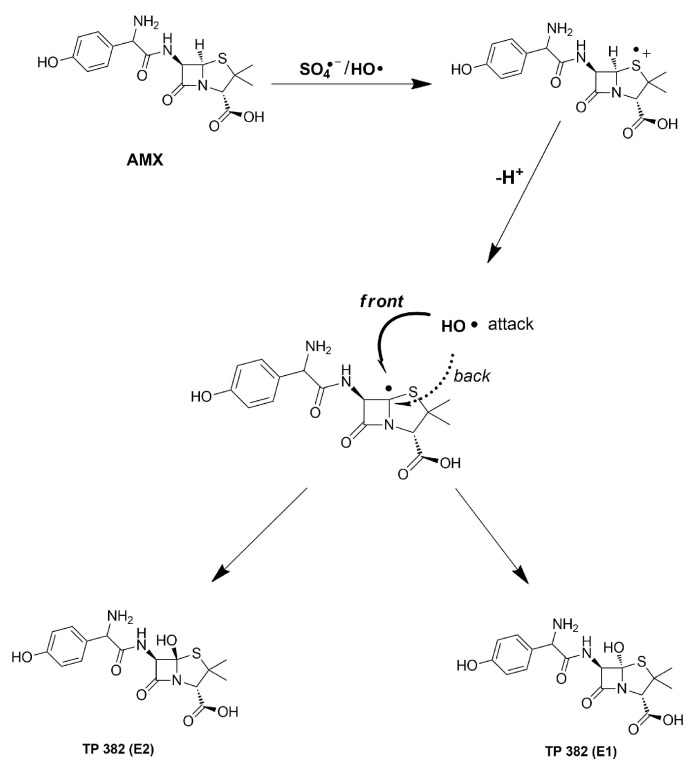
Proposed mechanism for the formation of TP 382 (E1 and E2).

Three oxidation TPs (TP 382 (S–O), TP 382 (E1), and TP 382 (E2)) were detected in both photocatalysis + H_2_O_2_ and photocatalysis + PS treatment processes. Accordingly, TP 382 (S–O) was formed via attack of SO_4_^•−^ and/or HO^•^ on the sulfur atom of the thioether moiety via an electron transfer mechanism, as confirmed by molecular orbital calculations [74]. TP 382 (E1) and TP 382 (E2) are ascribed to monohydroxylation of AMX. The AMX reaction centers that are susceptible to HO^•^ attack are illustrated in Figure 13. According to the MS2 results, hydroxylation on the methyl groups (C_3a_ and C_3b_) and aromatic ring (C_11–14_) was ruled out due to detection of fragments (*m*/*z*) 131.01610, and 107.04916, respectively (Figure S8). Moreover, the fragment proposed by Trovo et al., C_7_H_13_N_2_O_3_S (*m*/*z* = 189.0686), and other related fragments [73], which account for hydroxylation at the N_-8_ position (Figure 13), were not detected in this study. Instead, the *m*/*z* = 189.06583 fragment was detected, which was ascribed to C_10_H_9_N_2_O_2_, as proposed by Compound Discoverer^TM^ (Figure S8). Both SO_4_^•−^ and HO^•^ are expected to attack the sulfur atom of AMX to generate a sulfur-centered radical cation via an electron transfer mechanism [74]. Thereafter, this radical cation can be deprotonated to generate the α-thioether radical, which is susceptible to hydroxylation (Figure 14). As such, TP 382 (E1) and TP 382 (E2) are proposed since hydroxylation can occur on the positive/negative lobe of the α-thioether radical’s vacant p-orbital [80].

**Figure 15:**
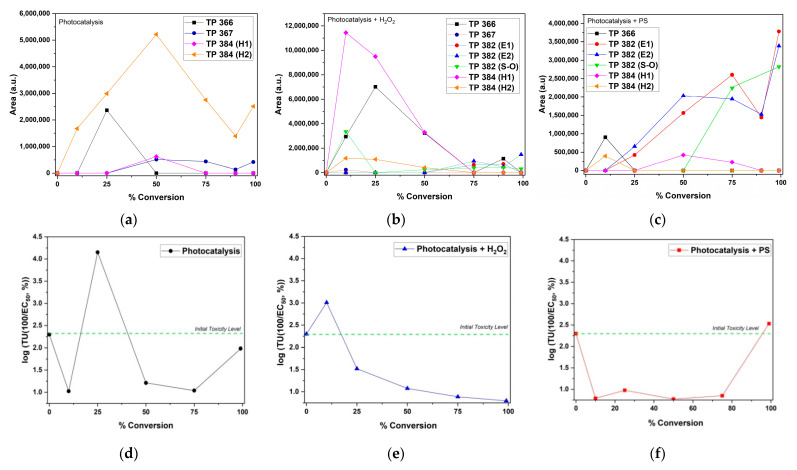
Evolution and conversion of identified AMX transformation byproducts for photocatalysis (**a**), photocatalysis + H_2_O_2_ (**b**), and photocatalysis + PS (**c**); corresponding toxicity toward *V. fischeri* (**d**–**f**).

The evolution and conversion profiles of TPs obtained from three different photocatalytic processes are presented in Figure 15a–c and correlated with toxicity profiles in Figure 15d–f, respectively. As can be seen in Figure 15a (photocatalysis), four byproducts were detected: TP 366, TP367, and the hydrolysis byproducts TP 384 (H1) and (H2). As compared to process toxicity profile (Figure 15d), it can observed that the sample reached the maximum 4.15 toxicity units (more toxic than initial level) at 25% AMX conversion. This result can be ascribed to TP 366 evolution, which also reached its maximum area at the same point (i.e., 25% AMX conversion). Specifically, TP 366 is amoxicillin 2′,5′-diketopiperazine, a known rearranged hydrolysis product of AMX, which has already been detected in Israel water effluents [75] and Spain river water samples [81]. Nevertheless, it must be noted that toxicity units dropped to 1.12 after reaching 50% AMX conversion, which also coincides with the decrease in TP 366 concentration. Although TP 384 (H2) is the dominant byproduct in the photocatalysis process, it had a minor contribution to the overall toxicity. TP 367 also had a minor contribution to overall toxicity, despite increased formation (50–99% AMX conversions extents). Clearly, the spike in toxicity units is directly linked to TP 366 formation.

As shown in Figure 15b (photocatalysis+ H_2_O_2_), seven byproducts were detected: TP 366, TP 367, TP 382 (E1 and E2), TP 382 (S–O), and TP 384 (H1 and H2). As compared to the process toxicity profile (Figure 15e), it can be observed that the sample reached the maximum of 3.01 toxicity units (more toxic than initial level) at 10% AMX conversion. This result can be ascribed to combined toxicity of TP 382 (S–O) with TP 366 and TP 384 (H1). It must be noted that TP 382 (S–O) also reached its maximum area at the same point (i.e., 10% AMX conversion). As reported in the literature, TP 382 (S–O) was found to be a contributor to the overall toxicity on persulfate-treated AMX aqueous solution [9]. Accordingly, toxicity units dropped to 1.52 upon reaching 25% AMX conversion, which coincides with the decrease in TP 382 (S–O) concentration. The maximum of TP 366 was reached at 50% AMX conversion, exhibiting no abrupt effect on the toxicity of the sample. Such results may be ascribed to the “antagonistic” effect of other TPs, such as the presence TP 384 (H1), which may have eventually led to the reduced toxicity of TP 366.

In Figure 15c (photocatalysis + PS), six byproducts were detected: TP 366, TP 382 (E1) and (E2), TP 382 (S–O), and TP 384 (H1 and H2). As compared to the process toxicity profile (Figure 15f), it can observed that the sample reached the maximum of 2.53 toxicity units (more toxic than initial level) at <99% AMX conversion. This result can be ascribed to the increased formation of TP 382 (E1) and (E2), as well as TP 382 (S–O), which also reached their maximum concentrations at the same point (i.e., <99% AMX conversion). All remaining TPs (i.e., TP 366, TP 367, and TP 384 (H1 and H2)) showed no synergistic and/or antagonistic effect on the overall toxicity.

### 3.5. Stability Test

**Figure 16:**
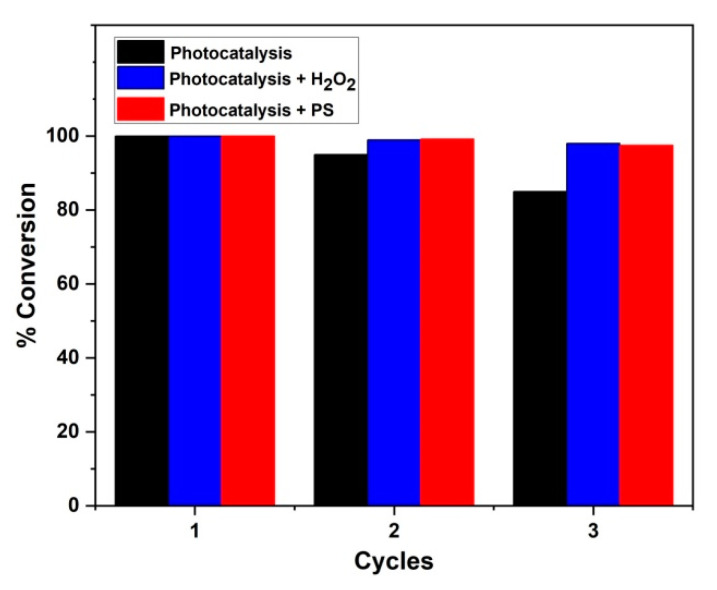
The %AMX conversion by photocatalysis, photocatalysis + H_2_O_2_, and photocatalysis + PS during three consecutive cycles. Experimental conditions: [AMX] = 0.05 mM; initial pH = 4.808; [PS] = [H_2_O_2_] = 1.873 mM; reaction time: photocatalysis (3900 min/cycle); photocatalysis + H_2_O_2_ (720 min/cycle); photocatalysis + PS (380 min/cycle); catalyst used in immobilized form.

Stability tests were performed for three consecutive cycles using the immobilized 5% Fe_2_O_3_/TiO_2_ photocatalyst with the optimum conditions obtained in Section 3.2. As shown in Figure 16, AMX conversion of <99% was achieved in three consecutive cycles of photocatalytic experiments containing PS and H_2_O_2_. However, 95% and 85% AMX conversions were achieved in the second and third cycles, respectively, of the sole photocatalysis process. The loss of activity of the immobilized photocatalyst during photocatalysis (*without oxidant*) in consecutive cycles was mainly due to overexposure (3900 min/cycle) compared to other processes containing PS and H_2_O_2_ (380 and 720 min/cycle, respectively).

## 4. Conclusions

Fe_2_O_3_/TiO_2_ nanocomposites were successfully prepared using an impregnation/calcination technique of TiO_2_-P25 and Fe(NO_3_)_3_·9H_2_O. XRD and RS analyses revealed that the obtained iron oxide was hematite, α-Fe_2_O_3_. Moreover, XRD, RS, XPS, and SEM/EDXS showed successful incorporation of α-Fe_2_O_3_ with TiO_2_. DRS results showed improved visible-light absorption and a decrease in overall bandgap values of Fe_2_O_3_/TiO_2_ nanocomposites upon increasing α-Fe_2_O_3_ content. Electrochemical experiments (EIS and photocurrent responses) revealed improved charge separation (*e^−^/h^+^)* of the obtained nanocomposite compared to its individual components (i.e., TiO_2_ and α-Fe_2_O_3_). Specifically, 5% (*w/w*) Fe_2_O_3_/TiO_2_ showed the highest photocatalytic activity based on preliminary photocatalytic experiments, as well as on the PL spectroscopy results. The results obtained from RSM modeling showed optimum conditions of [PS] = 1.873 mM and pH 4.808. Photocatalysis + PS achieved fastest AMX conversion, possessing a higher zero-order rate constant (*k* = 1.51 × 10^−7^ M·min^−1^) compared to photocatalysis + H_2_O_2_ (*k* = 1.11 × 10^−7^ M·min^−1^) and photocatalysis only (*k* = 0.35 × 10^−7^ M·min^−1^). ROS scavenging showed that photogenerated *h^+^* played the major role for AMX conversion in all processes. Toxicity changes of AMX solution were associated with TP 366 during photocatalysis, TP 382 (S–O) during photocatalysis + H_2_O_2_, and hydroxylated TPs (i.e., TP 382 (S–O) and TP 382 (E1 and E2)) during photocatalysis + PS. It is important to note that these AMX TPs greatly affected the toxicity of AMX solution during treatment in general.

## Figures and Tables

**Table 1:** Experimental range and levels of process/variables.

Process Parameters	Model Variables/Coded Values	Level/Range		
		−1	0	1
pH	*X*_1_	4	6	8
[S_2_O_8_^2−^] (µM)	*X*_2_	500	1750	3000

**Table 2:** Photocatalyst bandgap values estimated using Kubelka–Munk function.

Photocatalyst	TiO_2_ (P25)	1% Fe_2_O_3_/TiO_2_	3% Fe_2_O_3_/TiO_2_	5% Fe_2_O_3_/TiO_2_	10% Fe_2_O_3_/TiO_2_	20% Fe_2_O_3_/TiO_2_	α-Fe_2_O_3_
Bandgap (eV)	3.31	3.29	3.08	2.94	1.92	1.89	1.87

**Table 3:** Single-point and multipoint BET surface area of samples.

Photocatalyst	Single-Point BET (m^2^/g)	Multipoint BET (m^2^/g)
TiO_2_–P25	46.6112	47.3512 ± 0.1206
α-Fe_2_O_3_	25.2897	25.4548 ± 0.2408
1% Fe_2_O_3_/TiO_2_	49.7926	51.2769 ± 0.4552
3% Fe_2_O_3_/TiO_2_	39.3548	39.6042 ± 0.3677
5% Fe_2_O_3_/TiO_2_	40.4217	40.985 ± 0.2412
10% Fe_2_O_3_/TiO_2_	42.5486	43.4856 ± 0.1723
20% Fe_2_O_3_/TiO_2_	34.4068	34.7489 ± 0.3532

## Data Availability

The data presented in this study are available upon reasonable request from the corresponding author.

## Supplementary Materials

The following supporting information can be downloaded at https://www.mdpi.com/article/10.3390/nano12234328/s1: Figure S1: Images of TiO2 (P25) and Fe2O3/TiO2 nanocomposites; Figure S2: Full spectrum coverage of LED used in PEC tests; Figure S3: Photocatalytic removal of AMX using prepared 5% Fe2O3/TiO2 under visible-light irradiation with different PS concentration: (a) removal profile; (b) % removal per different PS concentration. Conditions: [catalyst dosage] = 0.5 g/L; [AMX] = 0.05 mM; initial pH = natural pH (5.5); Figure S4: Image of immobilized 5% Fe2O3/TiO2 nanocomposite onto glass support; Figure S5: AMX photocatalytic treatment by vis-(5% Fe2O3/TiO2)/PS. Experimental conditions set by FFD (Table S1); Figure S6: Residual diagnostics of RSM model predicting AMX removal rate constants (*kobs*) by vis-(5% Fe2O3/TiO2)/PS process after 150 min treatment: (A) observed vs. predicted plot, (B) normal probability plot, and (C) internally studentized residuals vs. predicted values plot; Figure S7: MS and MS2 spectra of AMX; Figure S8: MS and MS2 spectra of TP 382 (E1); Figure S9: MS and MS2 spectra of TP 382 (E2); Figure S10: MS and MS2 spectra of TP 382 (S–O); Figure S11: MS and MS2 spectra of TP 384 (H1); Figure S12: MS and MS2 spectra of TP 384 (H2); Figure S13: MS and MS2 spectra of TP 367; Figure S14: MS and MS2 spectra of TP 366; Figure S15. Formation of TP 365 (*m*/*z* = 365) and TP 367; Table S1: FFD matrix for AMX removal rate constants (*kobs*) by vis-(5% Fe2O3/TiO2)/PS process after 150 min treatment; Table S2: Analysis of variance (ANOVA) of RSM model predicting AMX removal rate constants (*kobs*) by vis-(5% Fe2O3/TiO2)/PS process after 150 min treatment; Table S3: Accurate mass measurements determined using LC–MS-Orbitrap of protonated AMX conversion products and their corresponding fragment ions. References [73,74,82,83,84] are cited in the supplementary materials
