# Supplementary material for: Visible-Light Activation of Persulfate or H2O2 by Fe2O3/TiO2 Immobilized on Glass Support for Photocatalytic Removal of Amoxicillin: Mechanism, Transformation Products, and Toxicity Assessment
Source: Nanomaterials (Basel). 2022 Dec 5;12(23):4328. doi: 10.3390/nano12234328 (PMC9738309; doi:10.3390/nano12234328)
Supplement: Supplementary file 1 [file nanomaterials-12-04328-s001.zip › nanomaterials-2068175-supplementary.pdf]

Supplementary Materials

Visible-Light Activation of Persulfate or H_2O_2 by Fe_2O_3/TiO_2 Immobilized on Glass Support for Photocatalytic Removal of Amoxicillin: Mechanism, Transformation Products, and Toxicity Assessment

Francis M. dela Rosa [1,2,3], Marin Popović [4], Josipa Papac Zjačić [1], Gabrijela Radić [1], Marijana Kraljić Roković [1], Marin Kovačić [1], María José Farré [2], Boštjan Genorio [5], Urška Lavrenčič Štangar [5], Hrvoje Kušić [1,*], Ana Lončarić Božić [1] and Mira Petrović [2,6]

1 Faculty of Chemical Engineering and Technology, University of Zagreb, Marulićev trg 19, 10000 Zagreb, Croatia
2 Catalan Institute for Water Research (ICRA), C/Emili Grahit, 101, 17003 Girona, Spain
3 Faculty of Sciences, University of Girona, 17071 Girona, Spain
4 Department of Safety and Protection, Karlovac University of Applied Sciences, Trg J.J. Strossmayera 9, 47000 Karlovac, Croatia
5 Faculty of Chemistry and Chemical Technology, University of Ljubljana, Vecna pot 113, 1000 Ljubljana, Slovenia
6 Catalan Institution for Research and Advanced Studies (ICREA), Passeig Lluis Companys 23, 08010 Barcelona, Spain
* Correspondence: hkusic@fkit.hr

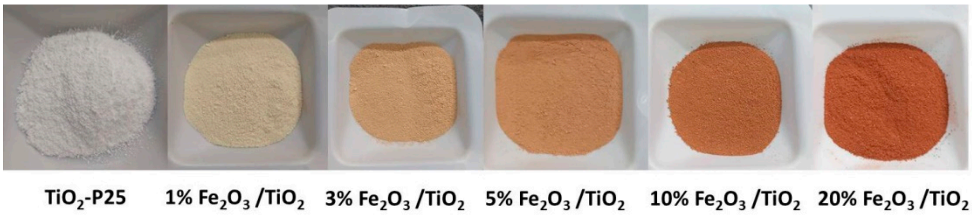

Figure S1. Photo images of TiO_2 (P25) and TiO_2/Fe_2O_3 nanocomposites

Table S1. FFD matrix for AMX removal rate constants (k_{obs}) by vis-(5% Fe_2O_3/TiO_2)/PS process after 150 min treatment

Exp. #	**Variables**		**Experimental results**	**Response, *Y***
	X_1	X_2		
	coded	coded	k_{obs}(AMX), × 10^{-7} M min^{-1}	
1	-1	-1	1.29	1.31
2	0	-1	1.27	1.23
3	1	-1	0.65	0.66
4	-1	0	1.48	1.46
5	0	0	1.40	1.41
6	1	0	0.87	0.87
7	-1	1	1.35	1.34
8	0	1	1.30	1.32
9	1	1	0.82	0.81

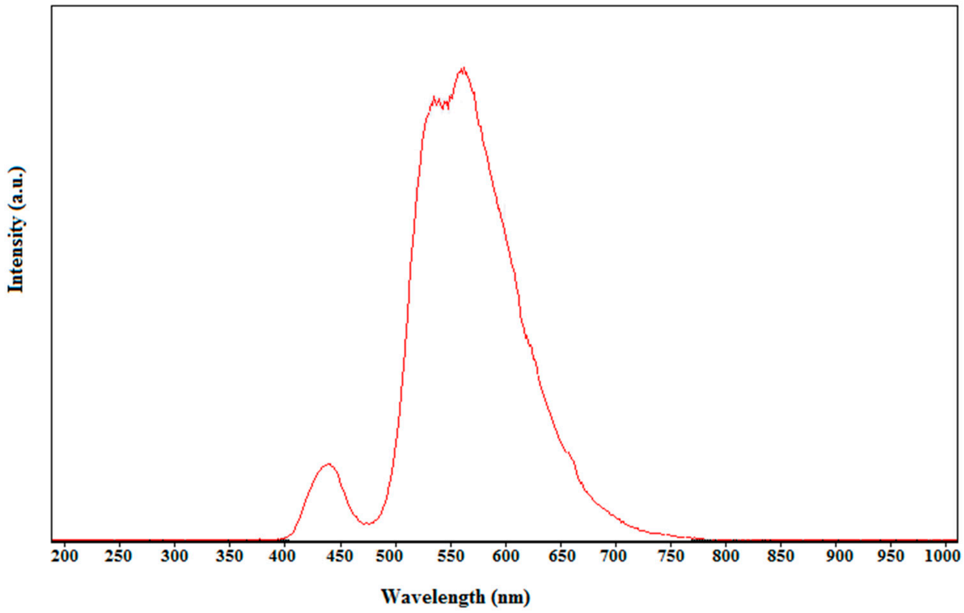

Figure S2. Full Spectrum Coverage of LED used in PEC tests

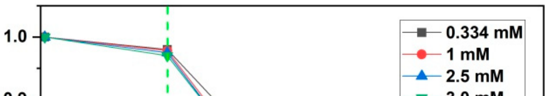

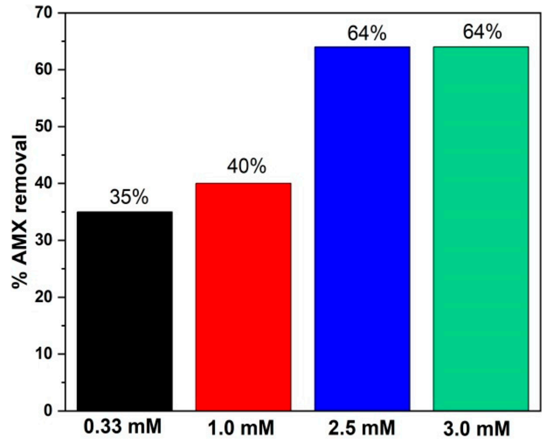

(a) **(b)**

Figure S3. Photocatalytic removal of AMX using prepared 5%Fe_2O_3/TiO_2 under visible light irradiation with different PS concentration; (a) removal profile and (b) % removal per different PS concentration(*Conditions:* [catalyst dosage]= 0.5 g/L ; [AMX]= 0.05 mM ; initial pH = natural pH (5.5))

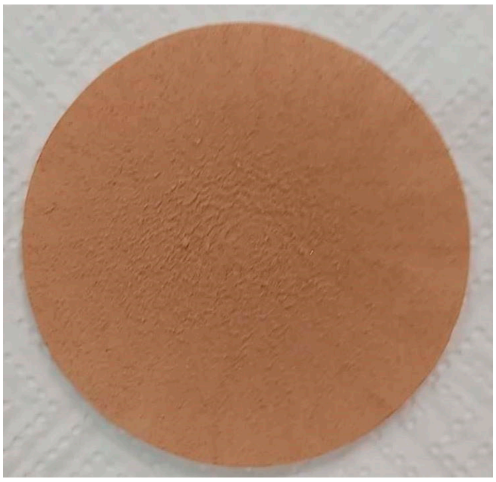

Figure S4. Photo image of immobilized 5% Fe_2O_3/TiO_2 nanocomposite onto glass support

Table S2. Analysis of variance (ANOVA) of RSM model predicting AMX removal rate constants (k_{obs}) by vis-(5% Fe_2O_3/TiO_2)/PS process after 150 min treatment

**Factor** (coded)	Statistical analysis				
	SS	df	MSS	F	p
Model	0.7014	5	0.1403	136.37	0.0010*
X_1	0.5281	1	0.5281	513.33	0.0002*
X_1^2	0.1217	1	0.1217	118.29	0.0017*
X_2	0.0113	1	0.0113	10.95	0.0454*
X_2^2	0.0374	1	0.0374	36.31	0.0092*
$X_1 \times X_2$	0.0030	1	0.0030	2.94	0.1849
Residual	0.0031	3	0.0010		
Total	0.7045	8			

*p<0.05 means that model or model term is significant

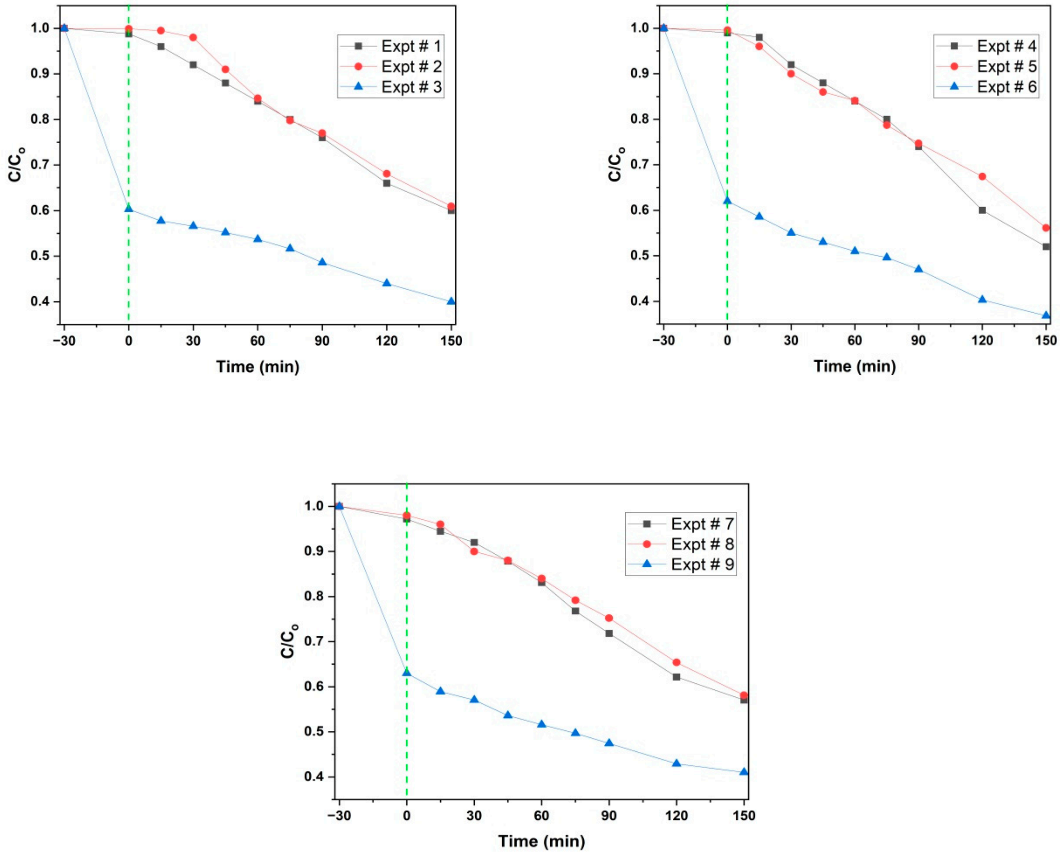

Figure S5. AMX photocatalytic treatment by vis-(5% Fe_2O_3/TiO_2)/PS. Experimental conditions set by FFD (Table S1, Supplementary material)

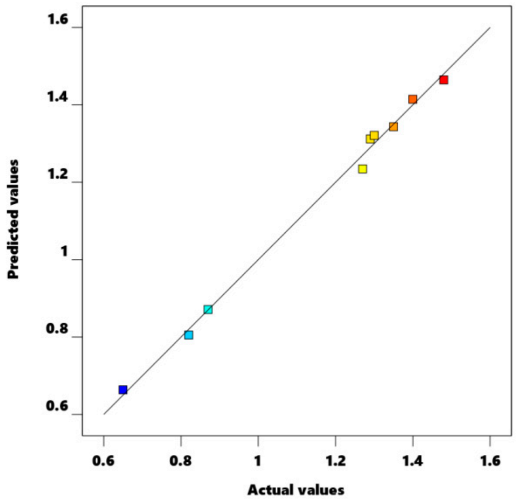

(**a**)

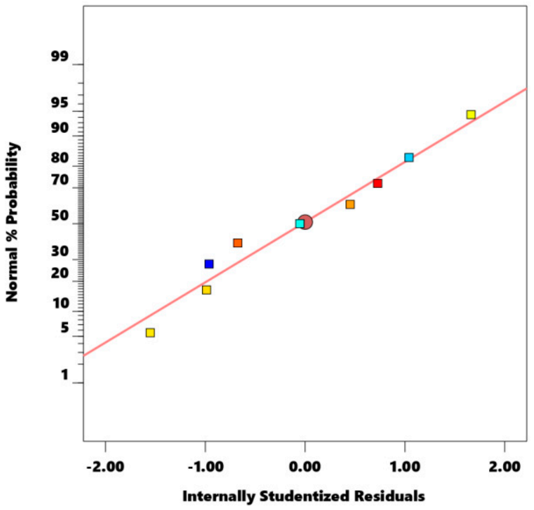

(**b**)

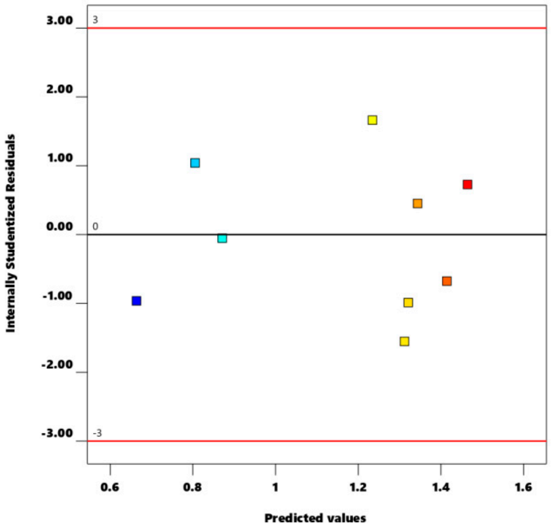

(**c**)

Figure S6. Residual diagnostics of RSM model predicting AMX removal rate constants (k_{obs}) by vis-(5% Fe_2O_3/TiO_2)/PS process after 150 min treatment: (A) observed *vs.* predicted plot, (B) normal probability plot, and (C) internally studentized residuals *vs.* predicted values plot

Table S3. Accurate mass measurements found by LC-MS-Orbitrap of protonated AMX conversion products and their corresponding fragment ions

R_t (min)	Compound	Chemical Formula	m/z (Experimental)	RDBE	Annotated Δmass (ppm)	FISh Coverage	Suggested chemical structure	Photocatalysis	Photocatalysis + H_2O_2	Photocatalysis + Persulfate	Reference
1.180	**AMX**	$C_{16}H_{20}N_3O_5S$ $[M+H]^+$	366.11102	8.5							[1,2]
		$C_{16}H_{17}N_2O_5S$ $[M+H–(NH_3)]^+$	349.08459	9.5							
		$C_{15}H_{17}N_2O_4S$	321.09079	8.5	-1.68	64.71					
		$C_{11}H_8NO_3S$	234.02155	8.5			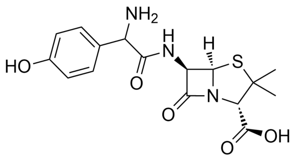	----	----	----	
		$C_{10}H_{10}NO_2S$	208.04260	6.5							
		$C_6H_{10}NO_2S$	160.04242	2.5							
		C_4H_4NOS	114.00060	3.5							
0.397	**TP 382**	$C_{16}H_{20}N_3O_6S$ $[M+H]^+$	382.10617	8.5	-						
	**(E1)**	$C_{16}H_{17}N_2O_6S$ $[M+H–(NH_3)]^+$	365.07965	9.5							Proposed
		$C_{10}H_9N_2O_2$	189.06583	7.5				X	✓	✓	Structure
		$C_9H_8NO_2$	162.05482	6.5	-1.48	43.75					
		$C_5H_{10}NO_2S$	148.04237	1.5			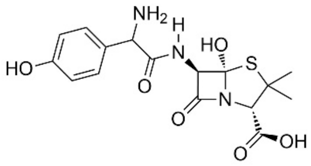				
		$C_5H_7O_2S$	131.01610	2.5							
		C_5H_5OS	113.00550	3.5							
		C_7H_7O	107.04916	4.5							
0.532	**TP 382**	$C_{16}H_{20}N_3O_6S$ $[M+H]^+$	382.10614	8.5	-						Proposed

	**(E2)**	$C_{16}H_{17}N_2O_6S$ [M+H–(NH$_3$)]$^+$	365.07974	9.5	-1.46	56.67					Structure
		$C_{10}H_9N_2O_2$	189.06616	7.5			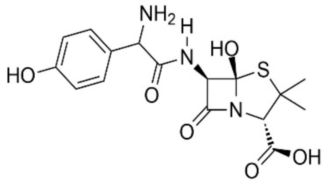	X	✓	✓	
		$C_9H_8NO_2$	162.05492	6.5							
		$C_5H_{10}NO_2S$	148.04259	1.5							
		$C_5H_7O_2S$	131.01564	2.5							
		C_5H_5OS	113.00517	3.5							
		C_7H_7O	107.04927	4.5							
0.929	**TP 382**	$C_{16}H_{20}N_3O_6S$ [M+H]$^+$	382.10602	8.5							[3][4,5]
	**(S-O)**	$C_{16}H_{17}N_2O_6S$ [M+H–(NH$_3$)]$^+$	365.07938	9.5							
		$C_{15}H_{17}N_2O_5$	337.08368	8.5	-1.44	100.00	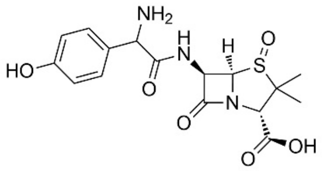	X	✓	✓	
		$C_9H_9N_2O$	161.07129	6.5							
1.106	**TP384 (H1)**	$C_{16}H_{22}N_3O_6S$ [M+H]$^+$	384.12177	7.5	-1.71	72.73					[2]
		$C_{16}H_{19}N_2O_6S$ [M+H–(NH$_3$)]$^+$	367.09509	8.5							
		$C_{15}H_{19}N_2O_4S$	323.10626	7.5			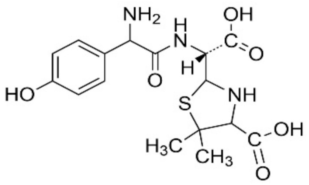	✓	✓	✓	
		$C_7H_{13}N_2O_4S$	189.06905	2.5							
		$C_6H_{10}NO_2S$	160.04237	2.5							
1.146	**TP384 (H2)**	$C_{16}H_{22}N_3O_6S$ [M+H]$^+$	384.12170	7.5	-1.90	80.00	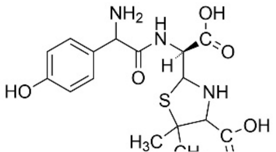				[2]

		$C_{16}H_{19}N_2O_6S$ [M+H–(NH$_3$)]$^+$	367.09537	8.5							
		$C_{15}H_{19}N_2O_4S$	323.10568	7.5				✓	✓	✓	
		$C_7H_{13}N_2O_4S$	189.06898	2.5							
		$C_6H_{10}NO_2S$	160.04245	2.5							
1.200	**TP367**	$C_{16}H_{19}N_2O_6S$ [M+H]$^+$	367.11459	7.5	-1.70	66.67					
		$C_{16}H_{17}N_2O_5S$ [M+H–(H$_2$O)]$^+$	349.08456	8.5							Proposed
		$C_{11}H_8NO_3S$	234.02122	8.5			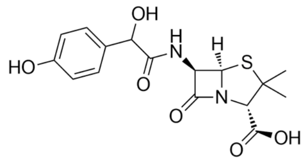	✓	✓	*X*	Structure
		$C_6H_{10}NO_2S$	160.04240	2.5							
		C_4H_4NOS	114.00066	3.5							
1.669	**TP366**	$C_{16}H_{20}N_3O_5S$ [M+H]$^+$	366.11102	8.5	-1.83	66.67					[2]
		$C_{10}H_{11}N_2O_3$	207.07625	6.5			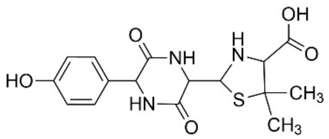				
		$C_6H_{10}NO_2S$	160.04257	2.5				✓	✓	✓	
		C_5H_8NS	114.03694	2.5							

References

[1] J.B. Arsand, R.B. Hoff, L. Jank, L.N. Meirelles, M. Silvia Díaz-Cruz, T.M. Pizzolato, D. Barceló, Transformation products of amoxicillin and ampicillin after photolysis in aqueous matrices: Identification and kinetics, Sci. Total Environ. 642 (2018) 954–967. https://doi.org/10.1016/j.scitotenv.2018.06.122.

[2] A.G. Trovó, R.F. Pupo Nogueira, A. Agüera, A.R. Fernandez-Alba, S. Malato, Degradation of the antibiotic amoxicillin by photo-Fenton process - Chemical and

toxicological assessment, Water Res. 45 (2011) 1394–1402. https://doi.org/10.1016/j.watres.2010.10.029.

[3] Y. Zhang, Y. Xiao, Y. Zhong, T.T. Lim, Comparison of amoxicillin photodegradation in the UV/H2O2 and UV/persulfate systems: Reaction kinetics, degradation pathways, and antibacterial activity, Chem. Eng. J. 372 (2019) 420–428. https://doi.org/10.1016/j.cej.2019.04.160.

[4] I. Gozlan, A. Rotstein, D. Avisar, Investigation of an amoxicillin oxidative degradation product formed under controlled environmental conditions, Environ. Chem. 7 (2010) 435–442. https://doi.org/10.1071/EN10037.

[5] I. Gozlan, A. Rotstein, D. Avisar, Amoxicillin-degradation products formed under controlled environmental conditions: Identification and determination in the aquatic environment, Chemosphere. 91 (2013) 985–992. https://doi.org/10.1016/j.chemosphere.2013.01.095.

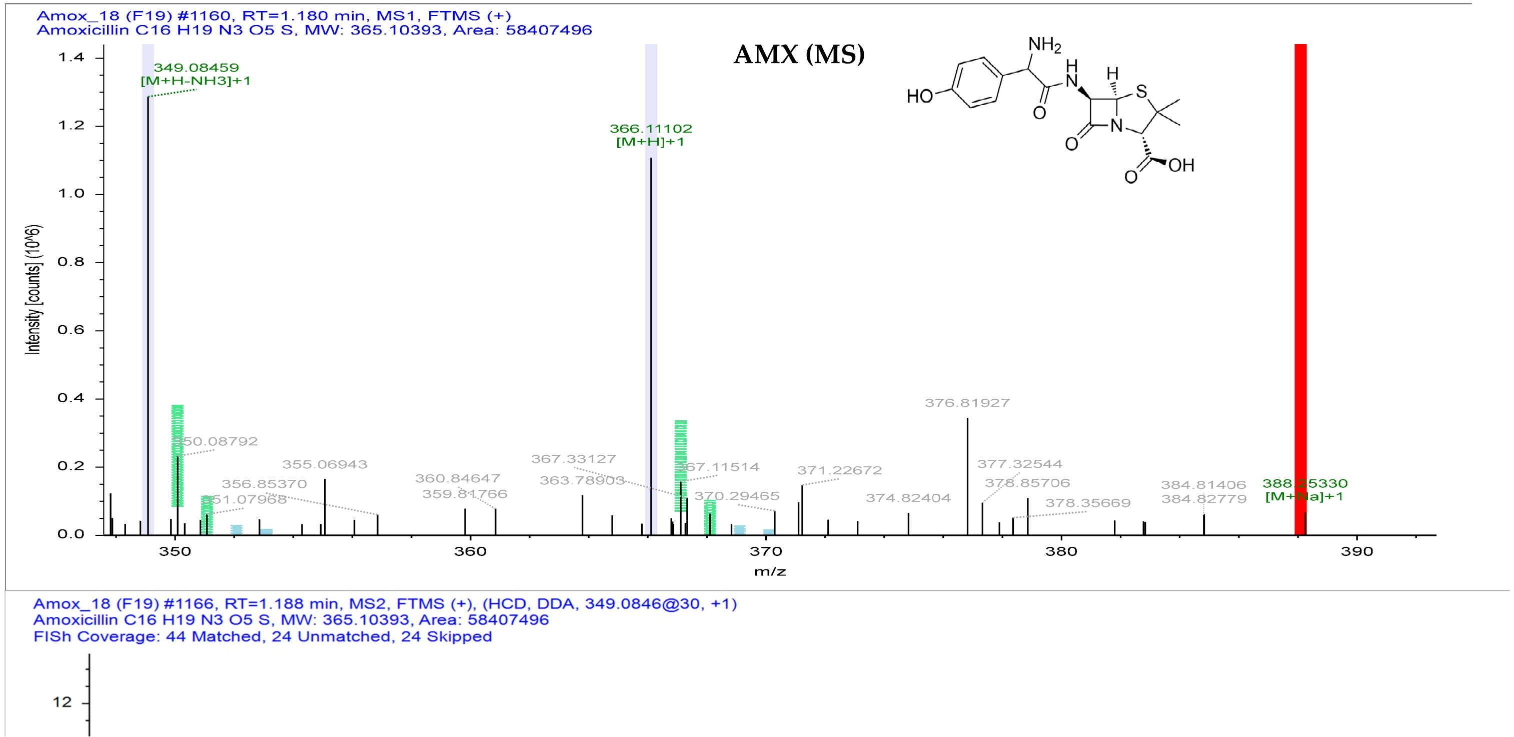

AMX (MS 2)

Figure S7. MS and MS spectra of AMX

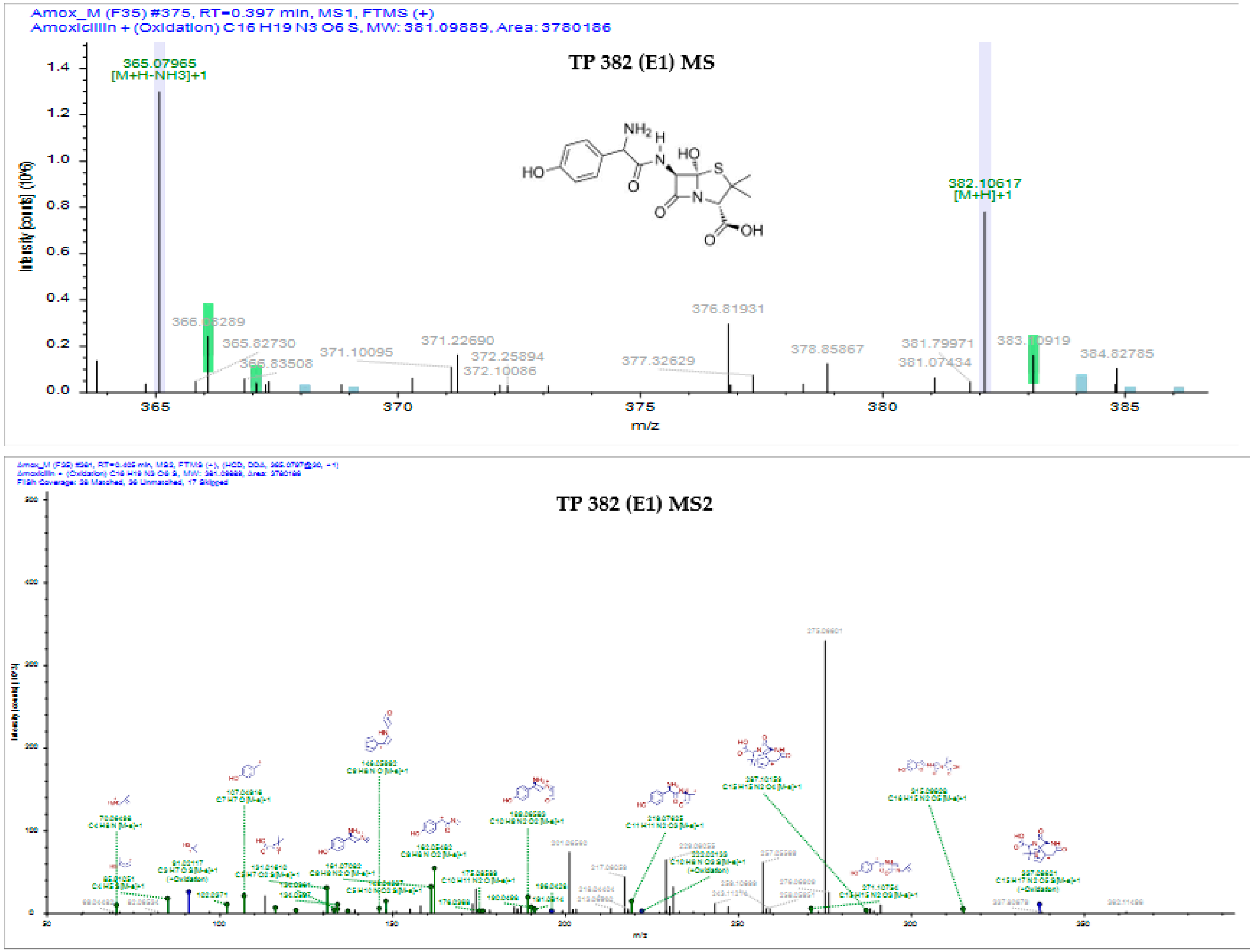
Amox_M (F35) #375, RT=0.397 min, MS1, FTMS (+)
Amoxicillin + (Oxidation) C16 H19 N3 O6 S, MW: 381.09889, Area: 3780186
TP 382 (E1) MS
365.07965
[M+H-NH3]+1
382.10617
[M+H]+1
366.08289
365.82730
366.83508
371.10095
371.22690
372.25894
372.10086
376.81931
377.32629
378.85867
381.79971
381.07434
384.82785
Intensity [counts] (10^6)
m/z
TP 382 (E1) MS2

(a)

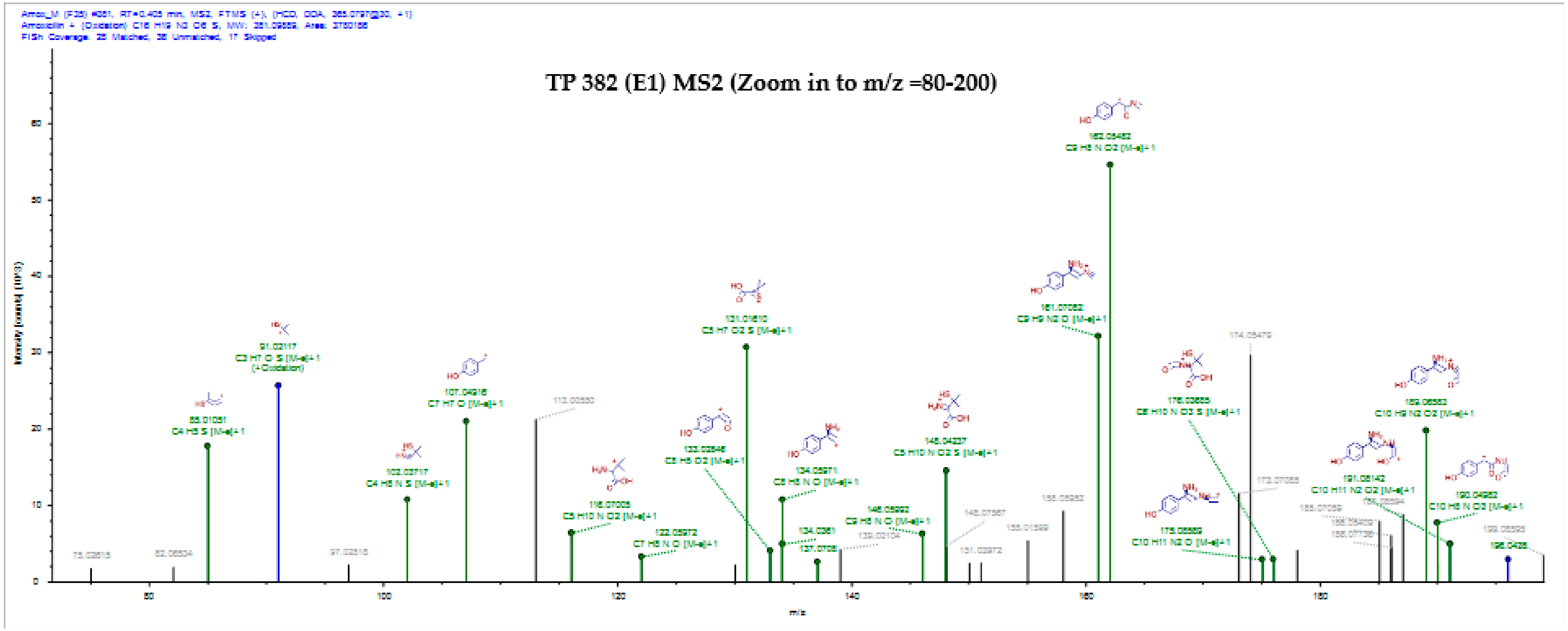

(**b**)

Figure S8. (a)MS and MS spectra of TP 382 (E1); (b) MS2 spectrum (magnified) of TP 382 (E1)

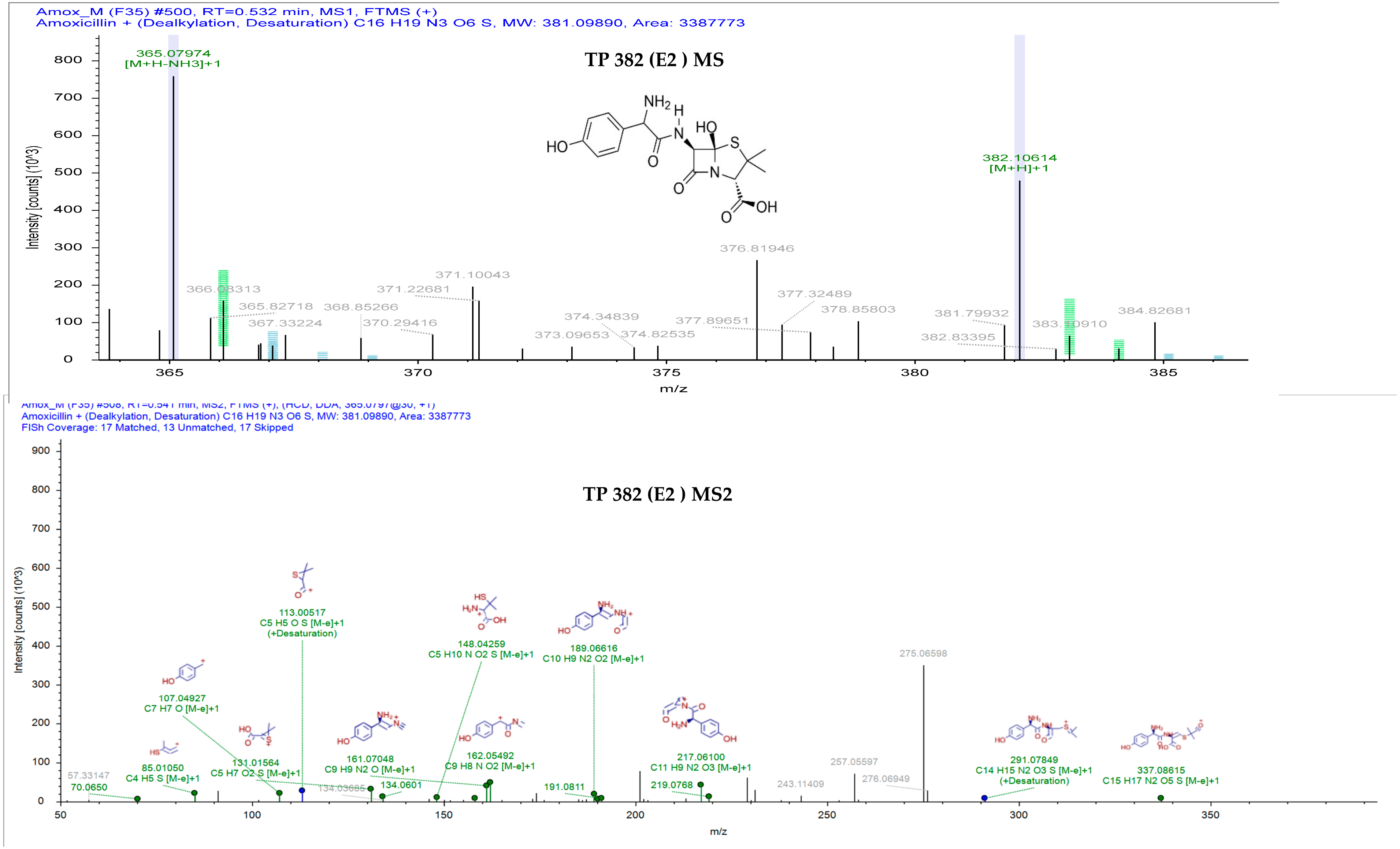

Figure S9. MS and MS2 spectra of TP 382 (E2)

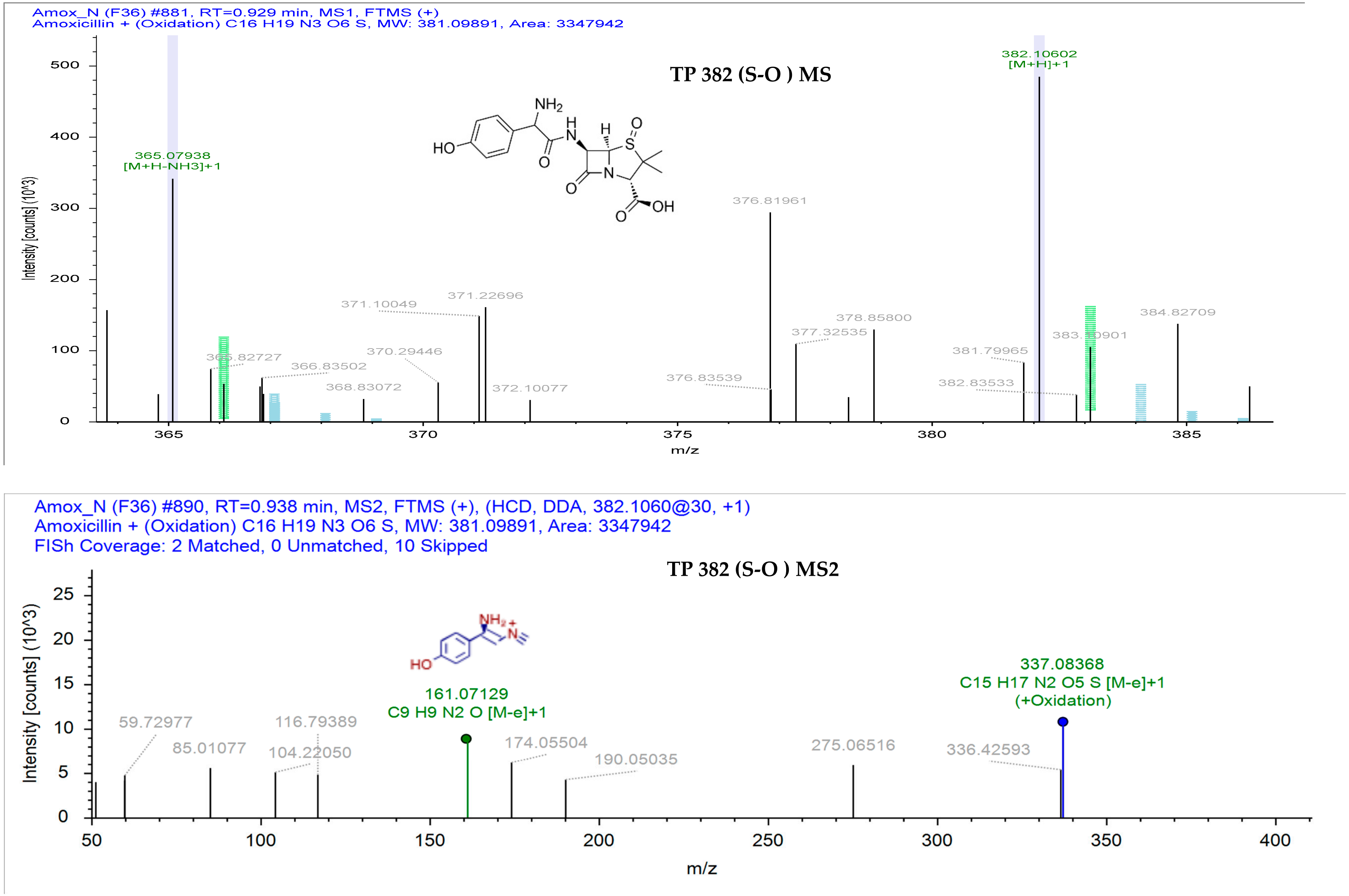

Figure S10. MS and MS2 spectra of TP 382 (S-O)

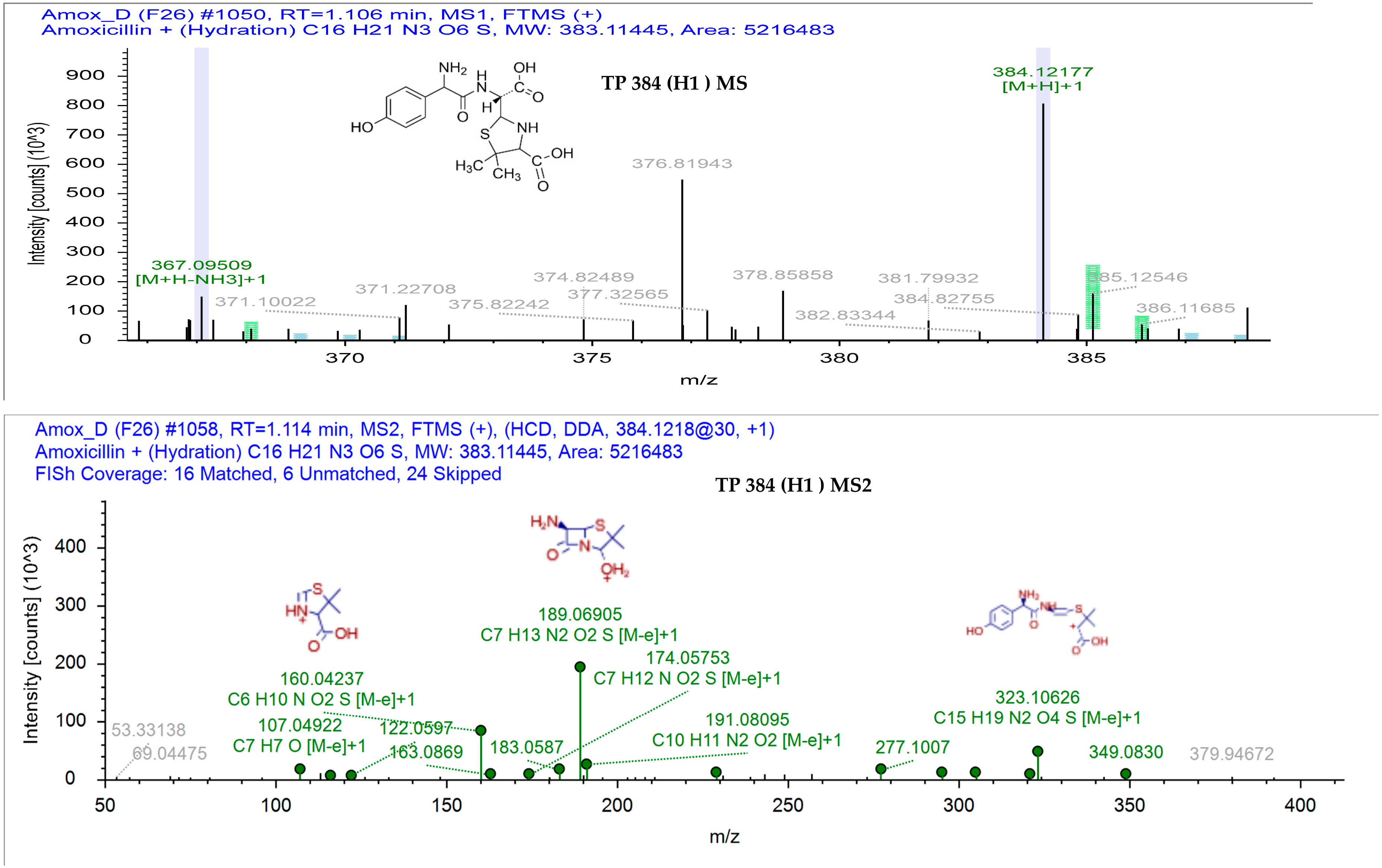

Figure S11. MS and MS2 spectra of TP 384 (H1)

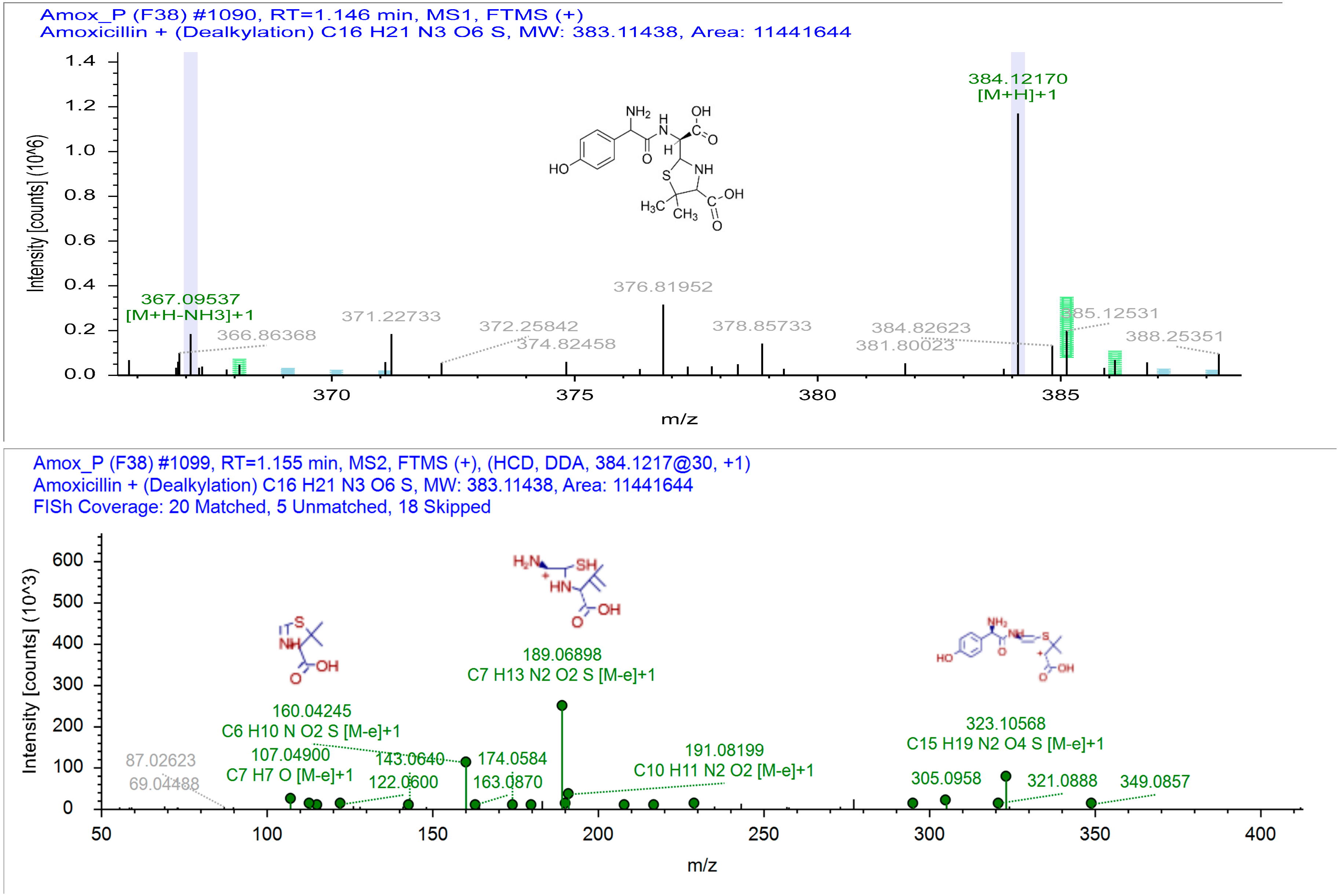
Amox_P (F38) #1090, RT=1.146 min, MS1, FTMS (+)
Amoxicillin + (Dealkylation) C16 H21 N3 O6 S, MW: 383.11438, Area: 11441644
Intensity [counts] (10^6)
1.4
1.2
1.0
0.8
0.6
0.4
0.2
0.0
384.12170
[M+H]+1
367.09537
[M+H-NH3]+1
366.86368
371.22733
372.25842
374.82458
376.81952
378.85733
384.82623
381.80023
385.12531
388.25351
370
375
380
385
m/z
Amox_P (F38) #1099, RT=1.155 min, MS2, FTMS (+), (HCD, DDA, 384.1217@30, +1)
Amoxicillin + (Dealkylation) C16 H21 N3 O6 S, MW: 383.11438, Area: 11441644
FISh Coverage: 20 Matched, 5 Unmatched, 18 Skipped
Intensity [counts] (10^3)
600
500
400
300
200
100
0
189.06898
C7 H13 N2 O2 S [M-e]+1
160.04245
C6 H10 N O2 S [M-e]+1
107.04900
C7 H7 O [M-e]+1
143.0640
174.0584
122.0600
163.0870
87.02623
69.04488
191.08199
C10 H11 N2 O2 [M-e]+1
323.10568
C15 H19 N2 O4 S [M-e]+1
305.0958
321.0888
349.0857
50
100
150
200
250
300
350
400
m/z
